# CDCA4 Promotes Lipid Metabolism in Triple-Negative Breast Cancer Through Activation of the SESN2/mTOR/SREBP1 Pathway

**DOI:** 10.3390/cancers18152517

**Published:** 2026-08-06

**Authors:** Jia Qi, Ming Cai, Xiaowen Wang, Peng Zhang, Jiani Wang, Jiezhong Wu, Weiling Huang, Wenxuan Wu, Kunpeng Hu, Xiaoyuan Liang

**Affiliations:** 1 Department of Breast and Thyroid Surgery, The Third Affiliated Hospital of Sun Yat-Sen University, Guangzhou 510700, China; qijia5@mail.sysu.edu.cn (J.Q.); wangxw68@mail3.sysu.edu.cn (X.W.);; 2 Division of Life Sciences and Medicine, Department of General Surgery, The First Affiliated Hospital of USTC, University of Science and Technology of China, Hefei 230001, China12118476@zju.edu.cn (W.W.); 3 Department of Breast Surgery, Aviation General Hospital, Beijing 100012, China

**Keywords:** triple-negative breast cancer, CDCA4, lipid metabolism, mTOR signaling

## Abstract

Triple-negative breast cancer (TNBC) is an aggressive subtype lacking effective targeted therapies. The molecular drivers of its progression and metabolic reprogramming remain unclear. Here, we identify cell division cycle-associated 4 (CDCA4) as a novel oncogenic driver in TNBC. Analysis of the TCGA-BRCA dataset, including 1085 breast cancer tissues and 112 normal tissues, showed that CDCA4 expression was significantly upregulated in breast cancer tissues. Subgroup analysis of TCGA-BRCA samples further showed higher CDCA4 expression (fold change = 1.707) in TNBC than in non-TNBC samples [TNBC, n = 116; non-TNBC, n = 984]. Survival analysis demonstrated that high CDCA4 expression was associated with poorer overall survival, with a hazard ratio of 1.54 (log-rank *p* = 0.0053). Functional assays demonstrated that CDCA4 knockdown suppresses proliferation, migration, invasion, and tumor growth in vitro and in vivo, whereas overexpression exerts opposite effects. RNA-sequencing revealed that CDCA4-regulated genes are enriched in lipid metabolism and mTOR signaling pathways. Mechanistically, CDCA4 depletion reduces intracellular lipids and the expression of lipogenic enzymes (FASN, ACC1). We show that CDCA4 activates mTOR and increases the nuclear active form of SREBP1, enhancing its promoter occupancy. Pharmacological mTOR inhibition reverses CDCA4-induced malignancy and metabolic alterations. Furthermore, Our findings suggest that SESN2 may contribute to CDCA4-mediated activation of mTOR signalling. SESN2 knockdown attenuates mTOR signaling and negates the pro-tumorigenic effects of CDCA4 overexpression. Collectively, these findings demonstrate that CDCA4 drives TNBC progression and lipid reprogramming via the SESN2/mTOR/SREBP1 axis, positioning CDCA4 as a potential prognostic biomarker and therapeutic target.

## Simple Summary

In this study, we found that CDCA4 is highly expressed in triple-negative breast cancer and is associated with poor patient prognosis. Functional experiments showed that CDCA4 is involved in regulating lipid metabolism. Mechanistically, CDCA4 may activate the mTOR/SREBP1 signaling pathway, thereby increasing lipid synthesis in triple-negative breast cancer cells. Our findings also suggest that SESN2 may contribute to CDCA4-mediated activation of mTOR signaling.

## 1. Introduction

Triple-negative breast cancer (TNBC) is a highly aggressive subtype of breast cancer defined by the absence of estrogen receptor, progesterone receptor, and human epidermal growth factor receptor 2 expression [1,2]. It accounts for approximately 10–15% of all breast cancers and is characterized by marked molecular heterogeneity, a high risk of early recurrence and distant metastasis, and relatively poor clinical outcomes compared with other breast cancer subtypes [3,4]. Although treatment strategies for TNBC have expanded in recent years to include immune checkpoint inhibitors, PARP inhibitors, and antibody–drug conjugates in selected clinical settings, chemotherapy remains the backbone of systemic treatment for a substantial proportion of patients [5,6]. The lack of broadly applicable molecular targets continues to be a major obstacle in TNBC management, underscoring the need to identify novel oncogenic drivers and therapeutically actionable pathways.

Cell division cycle-associated 4 (CDCA4), also known as HEPP/SEI-3, is a cell cycle-related nuclear protein implicated in the regulation of E2F-dependent transcription and cell proliferation through the E2F/RB axis [7]. Database annotation and earlier mechanistic studies have suggested that CDCA4 participates in cell-cycle progression, transcriptional regulation, and mitotic processes [8,9]. In our preliminary screening of public breast cancer datasets, we aimed to identify candidate genes that were aberrantly expressed in breast cancer, dysregulated in TNBC, and associated with shorter overall survival in patients with TNBC. Among the screened candidates, CDCA4 showed abnormal expression in breast cancer and TNBC, and its dysregulated expression was associated with poor overall survival in TNBC patients, supporting its potential clinical relevance.

Previous studies have reported that CDCA4 is aberrantly expressed in multiple human malignancies and may exert protumorigenic functions by promoting proliferation and survival signaling [10,11,12]. In breast cancer, CDCA4 knockdown has been shown to inhibit the proliferation of TNBC MDA-MB-231 cells in vitro and suppress xenograft tumor growth in vivo [9]. Another study suggested that CDCA4 may participate in TNBC progression as a downstream effector of the STAT1/circIFI30-related regulatory axis [13]. However, despite these findings, studies focusing on CDCA4 in TNBC remain limited, and the downstream mechanisms by which CDCA4 contributes to TNBC progression are still insufficiently defined.

Metabolic reprogramming is now recognized as a hallmark of cancer, and dysregulated lipid metabolism has emerged as a critical determinant of breast cancer progression [14]. Beyond serving as structural components of cellular membranes, lipids provide energy, signaling intermediates, and biosynthetic substrates that support rapid tumor growth, invasion, metastasis, and therapy resistance [15]. In breast cancer, increased de novo lipogenesis and intracellular lipid accumulation are frequently associated with aggressive phenotypes, and lipogenic regulators such as sterol regulatory element-binding protein 1 (SREBP1), fatty acid synthase (FASN), and acetyl-CoA carboxylase 1 (ACC1) have been implicated in disease progression [16,17]. Recent studies and reviews further highlight that metabolic heterogeneity, including altered lipid metabolism, is particularly relevant to TNBC biology [18,19].

Among the signaling networks that couple oncogenic cues to metabolic remodeling, the PI3K/AKT/mTOR pathway is one of the most extensively studied in breast cancer and TNBC [20,21,22]. Aberrant activation of this pathway promotes tumor cell proliferation, survival, and therapeutic resistance, while also coordinating anabolic metabolism [21]. mTOR signaling has been shown to regulate lipogenesis at least in part by promoting SREBP1 activation, thereby enhancing the transcription of downstream lipid synthesis genes [23,24]. Given the central role of mTOR in integrating growth factor signaling and metabolic control, elucidating upstream molecules that drive mTOR-dependent lipid metabolic reprogramming may provide mechanistic insight as well as potential therapeutic opportunities in TNBC [25].

In the present study, we identified CDCA4 as a highly expressed gene in TNBC and found that elevated CDCA4 was associated with poor prognosis. Functional analyses demonstrated that CDCA4 promoted TNBC cell proliferation, migration, invasion, and xenograft tumor growth. Transcriptomic and biochemical analyses further indicated that CDCA4 was involved in lipid metabolic reprogramming, as evidenced by altered intracellular lipid accumulation and lipogenic enzyme expression. Mechanistically, CDCA4 activated the mTOR/SREBP1 signaling axis to enhance lipogenic transcription, while inhibition of mTOR largely reversed the malignant phenotypes and lipid metabolic alterations induced by CDCA4. Moreover, we identified SESN2 as a mediator linking CDCA4 to mTOR activation. Collectively, our findings reveal a previously unrecognized CDCA4/SESN2/mTOR/SREBP1 regulatory axis in TNBC and suggest that targeting this pathway may represent a potential therapeutic strategy for this aggressive breast cancer subtype.

## 2. Materials and Methods

### 2.1. Bioinformatics Analysis of CDCA4 Expression and Survival

CDCA4 mRNA expression data and corresponding clinical information were obtained from The Cancer Genome Atlas Breast Invasive Carcinoma dataset (TCGA-BRCA). The expression values used in this study were RSEM-normalized expression values or TPM values derived from TCGA processed RNA-seq data. CDCA4 expression was first compared between breast cancer tumor tissues and normal breast tissues. For subtype analysis, breast cancer samples were classified into TNBC and non-TNBC groups according to the available receptor status annotations. Overall survival analysis was performed in TNBC patients from the TCGA cohort after stratification into CDCA4-high and CDCA4-low groups based on CDCA4 expression levels.

### 2.2. Cell Culture and Transfection

Human TNBC cell lines MDA-MB-468, HCC1937, and MDA-MB-231, as well as the normal mammary epithelial cell line MCF10A, were used in this study. All cell lines were obtained from the National Collection of Authenticated Cell Cultures, Chinese Academy of Sciences. MDA-MB-468, HCC1937, and MDA-MB-231 cells were cultured in DMEM (Invitrogen) supplemented with 10% fetal bovine serum (FBS; Life Technologies). MCF10A cells were cultured in MEGM medium (Lonza, Cat. No. CC-3150) supplemented with cholera toxin at a final concentration of 100 ng/mL. All cells were maintained in a humidified incubator containing 5% CO_2_ at 37 °C.

For transient transfection, cells were seeded in appropriate culture plates and transfected with siRNAs or plasmids using Lipofectamine™ 2000 (Invitrogen) according to the manufacturer’s instructions. The amount of siRNAs used for transfection was 30 pmol per 3.5 cm dish, and the amount of plasmid used was 2 μg per 3.5 cm dish. Cells were harvested 24–48 h after transfection for subsequent experiments.

### 2.3. Rapamycin Treatment

To inhibit mTOR signaling, TNBC cells were treated with rapamycin as an mTOR pathway inhibitor. For rescue experiments, CDCA4-overexpressing cells were treated with 1 μM rapamycin (MCE HY-10219) for 24 h before subsequent functional assays and molecular analyses.

### 2.4. siRNA Synthesis and Gene Silencing

Small interfering RNAs (siRNAs) targeting CDCA4 and SESN2, as well as the corresponding negative control siRNAs, were synthesized by Shanghai Biosciences. Cells were transfected with siRNAs using Lipofectamine™ 2000 (Invitrogen) according to the manufacturer’s protocol. Knockdown efficiency was assessed by RT-qPCR and/or Western blot analysis 24–48 h after transfection. The sequences of the siRNAs are listed in Supplementary Table S1.

### 2.5. RNA Extraction and RT-qPCR

Total RNA was extracted from cultured cells using a standard RNA isolation procedure. Genomic DNA contamination was removed using RNase-free DNase I (Invitrogen) according to the manufacturer’s instructions. First-strand cDNA was synthesized using HiScript QRT SuperMix (Vazyme). Quantitative real-time PCR was performed using the SYBR Green Master Mix Kit (Vazyme) on a real-time PCR system. Relative mRNA expression levels were calculated using the 2^−ΔΔCt^ method with β-actin as the internal control. Primer sequences used in this study are listed in Supplementary Table S1.

### 2.6. Western Blot Analysis

Cells or tumor tissues were lysed in RIPA lysis buffer supplemented with protease and phosphatase inhibitors. Protein concentrations were determined using the BCA Protein Assay Kit (Beyotime). Equal amounts of protein were separated by SDS-PAGE and transferred onto PVDF membranes (Millipore). After blocking with 5% non-fat milk or bovine serum albumin, the membranes were incubated overnight at 4 °C with the indicated primary antibodies, followed by incubation with HRP-conjugated secondary antibodies. Protein signals were detected using the ECL-PLUS Kit (Amersham). Details of the primary antibodies, including catalog numbers and dilution ratios, are provided in Supplementary Table S1.

### 2.7. Colony Formation Assay

For the colony formation assay, transfected cells were seeded into 6-well plates at low density and cultured for 10–14 days. The medium was changed every 3–4 days. At the end of the incubation period, colonies were fixed with paraformaldehyde and stained with crystal violet. Colonies containing more than 50 cells were counted under a light microscope.

### 2.8. MTS Assay

Cell proliferation was assessed using the MTS assay. Briefly, transfected cells were seeded into 96-well plates at an appropriate density. At the indicated time points (0~4 days), MTS reagent was added to each well and incubated according to the manufacturer’s instructions. Absorbance was measured at 490 nm using a microplate reader.

### 2.9. EdU Incorporation Assay

Cell proliferation was further evaluated using an EdU incorporation assay. Briefly, transfected cells were incubated with EdU working solution for the indicated period, fixed with paraformaldehyde, permeabilized, and stained according to the manufacturer’s instructions. Nuclei were counterstained with DAPI. Images were captured under a fluorescence microscope, and the percentage of EdU-positive cells was quantified.

### 2.10. Transwell Migration and Invasion Assays

Cell migration and invasion were evaluated using Transwell inserts with 8-μm pore size. For the migration assay, transfected cells suspended in serum-free medium were seeded into the upper chamber, whereas medium containing 10% FBS was added to the lower chamber as a chemoattractant. After incubation for 8 h, cells remaining on the upper surface were removed, and migrated cells on the lower surface were fixed, stained, and counted under a microscope. For the invasion assay, the upper chambers were pre-coated with Matrigel before cell seeding, and the remaining procedures were performed as described for the migration assay. The relative migration or invasion rate was calculated by normalizing the number of migrated or invaded cells in each treatment group to that of the control group.

### 2.11. RNA-Sequencing and KEGG Pathway Enrichment Analysis

Total RNA was extracted from control and CDCA4-knockdown TNBC cells and subjected to RNA-sequencing by Majorbio Bio-pharm Technology Co., Ltd. Each group included three biological replicates. The reference species was Homo sapiens, and the reference genome version used for analysis was GRCh38. After quality control and filtering, clean reads were aligned to the reference genome using HISAT2. Gene expression levels were quantified, and differentially expressed genes (DEGs) between the control and CDCA4-knockdown groups were identified using DESeq2. DEGs were defined according to the cutoff criteria of |log2 fold change| > 1.5 and adjusted *p* < 0.05. Volcano plots and hierarchical clustering heatmaps were generated based on the identified DEGs. KEGG pathway enrichment analysis was subsequently performed to identify significantly enriched biological pathways associated with CDCA4-regulated genes, using an adjusted *p*-value < 0.05 as the significance threshold.

### 2.12. ChIP-qPCR Assay

Chromatin immunoprecipitation (ChIP) was performed using a standard protocol. Briefly, cells were cross-linked with formaldehyde, lysed, and sonicated to generate chromatin fragments of appropriate size. Chromatin samples were immunoprecipitated overnight at 4 °C with an antibody against SREBP1-N or normal IgG as a negative control. After reversal of cross-linking and DNA purification, the precipitated DNA was analyzed by qPCR using primers specific for the promoter regions of FASN and ACC1. Relative enrichment was normalized to input DNA.

### 2.13. Measurement of Lipid Accumulation-Related Indicators

Intracellular lipid metabolism-related indicators were assessed using both Oil Red O staining and commercial biochemical assay kits. For Oil Red O staining, cells were fixed and stained using the Oil Red O Staining Kit (Beyotime) according to the manufacturer’s instructions. Lipid accumulation was then evaluated by microscopic observation and image acquisition, and the Oil Red O-positive staining area was quantified using image analysis software.

Intracellular free fatty acid (FFA), total triglyceride (TG), and total cholesterol (TC) levels were quantified using the Free Fatty Acid Assay Kit (Beyotime), Total Triglyceride Assay Kit (Beyotime), and Total Cholesterol Assay Kit (Beyotime), respectively. Briefly, 5 × 10^6^ cells were collected for each sample and lysed in 200 μL of lysis buffer. After centrifugation, the supernatant was collected for absorbance measurement according to the manufacturers’ instructions. Standard solutions supplied with each kit were used to generate standard curves. The concentrations of FFA, TG, and TC in the cell lysates were calculated based on the corresponding standard curves.

### 2.14. Xenograft Tumor Assay

All animal experiments were reviewed and approved by the Institutional Animal Care and Use Committee of Guangzhou Institute of Biomedicine and Health, Chinese Academy of Sciences, under animal welfare approval number 2026006, with approval granted on 2 February 2026. Female BALB/c nude mice, aged 6 weeks and weighing approximately 20 g, were obtained from Zhuhai Bestest Biotechnology Co., Ltd. A total of 16 mice were used in this study. Mice were housed in the animal facility of the Chinese Academy of Sciences under SPF conditions, with free access to food and water and a 12-h light/dark cycle. Animals were acclimatized for 5–7 days before the experiment.

For the subcutaneous xenograft model, cells were cultured to the logarithmic growth phase, collected, washed, and resuspended in sterile PBS. Mice were randomly divided into two groups, with 8 mice in each group. A total volume of 100 μL cell suspension was injected subcutaneously into each mouse. Tumor growth was monitored beginning 4 days after inoculation and measured every 2–3 days using calipers. Tumor volume was calculated using the formula: volume = length × width^2^/2. General condition, food intake, activity, and local tumor status were monitored throughout the experiment.

Humane endpoints included tumor diameter > 15 mm, tumor volume > 1500 mm^3^, body weight loss > 20%, ulceration, bleeding, infection, impaired normal activity, or obvious signs of distress or weakness. Mice were euthanized by CO_2_ inhalation at the experimental endpoint or when humane endpoint criteria were reached. Tumor tissues were collected for subsequent analysis.

### 2.15. Statistical Analysis

All experiments were independently repeated at least three times, and data are presented as the mean ± standard deviation (SD). Statistical analyses were performed using GraphPad Prism 9.5. Comparisons between two groups were analyzed using Student’s *t*-test, whereas differences among multiple groups were analyzed using one-way analysis of variance (ANOVA) followed by appropriate post hoc tests. For RNA-seq analysis, multiple testing correction was performed, and adjusted *p*-values were used for the identification of differentially expressed genes. Kaplan–Meier survival analysis with the log-rank test was used to evaluate overall survival. A *p*-value < 0.05 was considered statistically significant.

## 3. Results

### 3.1. CDCA4 Is Upregulated in TNBC and Predicts Poor Clinical Outcome

**Figure 1:**
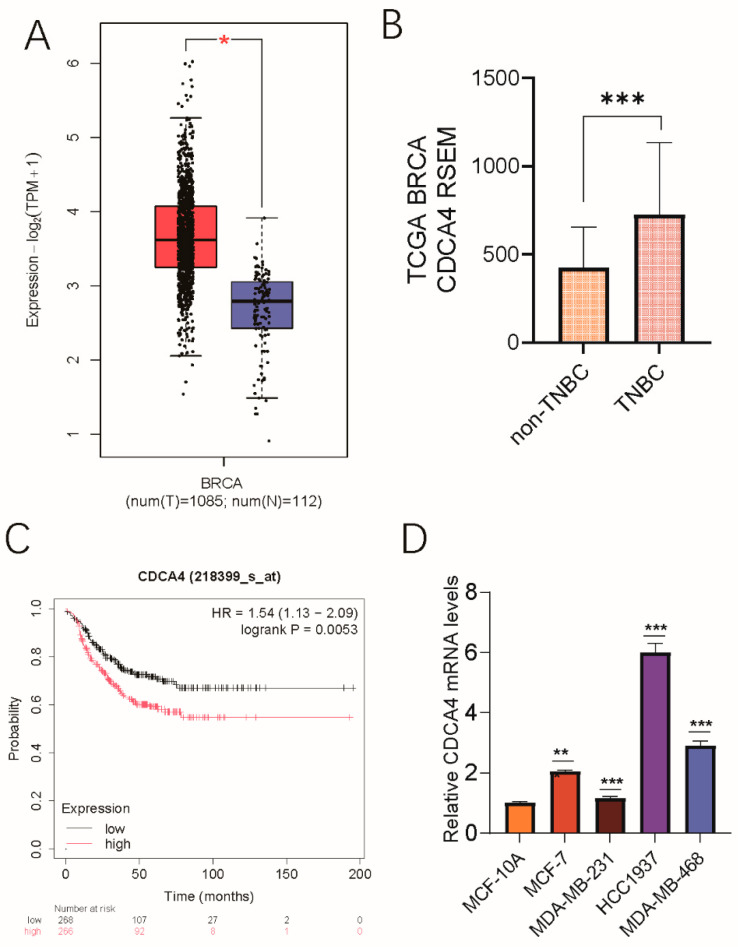
Analysis of CDCA4 expression and survival association in breast cancer tissues and cell lines. (**A**) Analysis of CDCA4 mRNA expression in tumor tissues versus normal tissues in the TCGA-BRCA dataset. (**B**) Analysis of CDCA4 expression in non-TNBC and TNBC samples from the TCGA-BRCA dataset using RSEM-normalized expression values. (**C**) Overall survival analysis of TNBC patients with high versus low CDCA4 expression in the TCGA dataset. (**D**) RT-qPCR analysis of CDCA4 expression in normal mammary epithelial cells and TNBC cell lines.

To identify candidate oncogenes involved in the initiation and progression of triple-negative breast cancer (TNBC), we first analyzed the TCGA-BRCA cohort. CDCA4 mRNA expression was significantly elevated in breast cancer tissues (1085 breast cancer tissues) compared with normal breast tissues (112 normal tissues) (Figure 1A). Further stratified analysis revealed that CDCA4 expression was markedly higher (fold change = 1.707) in TNBC samples than in non-TNBC samples (Figure 1B).

To determine the prognostic relevance of CDCA4 in TNBC, Kaplan–Meier survival analysis was performed in TNBC patients. A total of 536 TNBC patients were included in the survival analysis, and patients were divided into high- and low-CDCA4 expression groups according to the median cutoff value. The results showed that high CDCA4 expression was significantly associated with reduced overall survival, with a hazard ratio of 1.54 (log-rank *p* = 0.0053) (Figure 1C).

To further evaluate whether CDCA4 is an independent prognostic factor, we performed multivariate Cox regression analysis. The analysis showed that CDCA4 expression remained significantly associated with overall survival after adjustment for these clinical parameters (HR = 1.185, *p* < 0.001; Table S2). These results suggest that elevated CDCA4 expression is associated with poor prognosis and may serve as an independent prognostic factor in TNBC.

### 3.2. CDCA4 Promotes the Malignant Phenotypes of TNBC Cells

**Figure 2:**
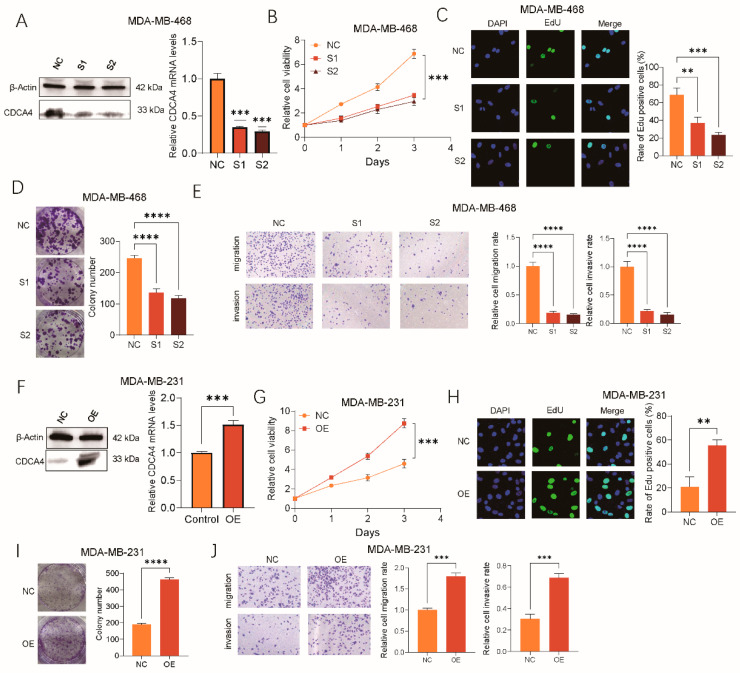
Functional analyses of CDCA4 knockdown and overexpression in TNBC cells. (**A**) Western blot and RT-qPCR analysis of CDCA4 expression in MDA-MB-468 cells after transfection with siRNAs targeting CDCA4. (**B**) MTS assay in CDCA4 knockdown MDA-MB-468 cells. (**C**) EdU incorporation assay in CDCA4 knockdown MDA-MB-468 cells. (**D**) Colony formation assay in CDCA4 knockdown MDA-MB-468 cells. (**E**) Transwell migration and invasion assays in CDCA4 knockdown MDA-MB-468 cells. (**F**) Western blot and RT-qPCR analysis of CDCA4 expression in MDA-MB-231 cells after CDCA4 overexpression. (**G**) MTS assay in CDCA4 -OE MDA-MB-231 cells. (**H**) EdU incorporation assay in CDCA4 -OE MDA-MB-231 cells. (**I**) Colony formation assay in CDCA4 -OE MDA-MB-231 cells. (**J**) Transwell migration and invasion assays in CDCA4 -OE MDA-MB-231 cells.

To further investigate the biological functions of CDCA4 in TNBC cells, loss- and gain-of-function assays were performed in different TNBC cell lines according to their endogenous CDCA4 expression levels. CDCA4 was silenced in MDA-MB-468 and HCC1937 cells, which exhibited relatively high CDCA4 expression, whereas CDCA4 was ectopically overexpressed in MDA-MB-231 cells, which expressed relatively low levels of CDCA4. Two siRNAs targeting CDCA4 (si-CDCA4-S1 and si-CDCA4-S2) were designed and transfected into MDA-MB-468 and HCC1937 cells. Western blot and RT-qPCR analysis confirmed that both siRNAs efficiently reduced CDCA4 expression in MDA-MB-468 cells and HCC1937 cells (Figure 2A and Figure S1A).

Functional assays were subsequently performed to evaluate the effect of CDCA4 knockdown on TNBC cell behavior. MTS assays showed that silencing CDCA4 markedly suppressed cell viability in MDA-MB-468 and HCC1937 cells (Figure 2B and Figure S1B). Consistently, EdU incorporation assays and colony formation assays demonstrated that CDCA4 knockdown significantly impaired the proliferative capacity of both cell lines (Figure 2C,D and Figure S1C,D). In addition, Transwell migration and invasion assays revealed that depletion of CDCA4 attenuated the migratory and invasive abilities of MDA-MB-468 and HCC1937 cells (Figure 2E and Figure S1E).

To further validate the oncogenic role of CDCA4 in TNBC, gain-of-function experiments were performed in MDA-MB-231 cells. Western blot and RT-qPCR confirmed the overexpression efficiency of CDCA4 following plasmid transfection (Figure 2F). MTS, EdU, and colony formation assays showed that CDCA4 overexpression enhanced the proliferative capacity of MDA-MB-231 cells (Figure 2G–I). Moreover, Transwell assays demonstrated that CDCA4 overexpression promoted the migration and invasion of MDA-MB-231 cells (Figure 2J). Collectively, these results indicate that CDCA4 facilitates TNBC cell proliferation, migration, and invasion.

### 3.3. CDCA4 Regulates Lipid Metabolic Reprogramming in TNBC Cells

**Figure 3:**
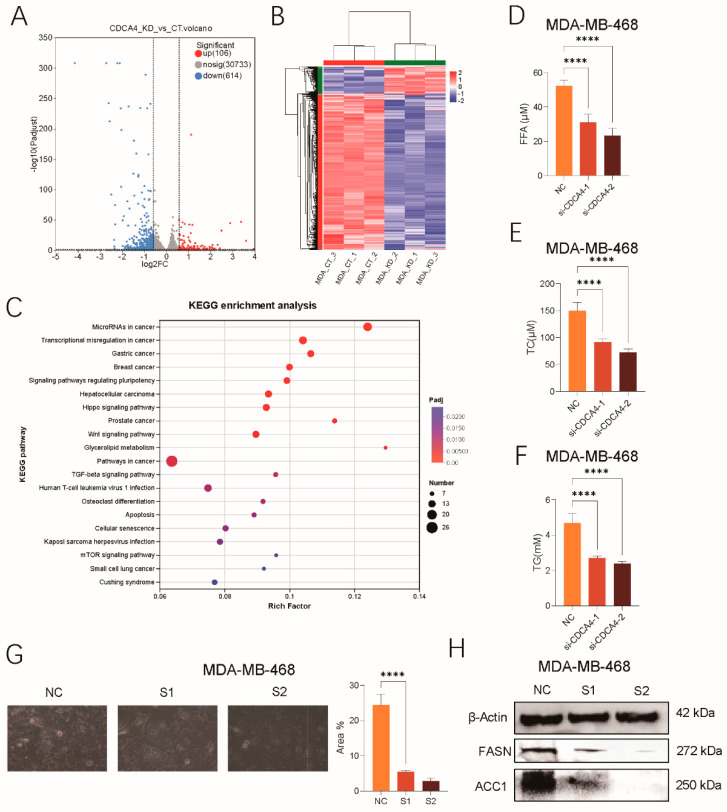
Transcriptomic and lipid metabolic analyses in MDA-MB-468 cells after CDCA4 knockdown. (**A**) Volcano plot of differentially expressed genes identified by RNA-seq analysis after CDCA4 knockdown. (**B**) Heatmap of hierarchical clustering analysis based on differentially expressed genes in control and CDCA4-knockdown cells. (**C**) KEGG pathway enrichment analysis of differentially expressed genes after CDCA4 knockdown. (**D**–**G**) Quantification of intracellular free fatty acid (FFA) levels (**D**), total cholesterol (TC) content (**E**), triglyceride (TG) content (**F**), and Oil Red O staining (**G**) in control and CDCA4-knockdown MDA-MB-468 cells. (**H**) Western blot analysis of FASN and ACC1 protein expression in control and CDCA4-knockdown MDA-MB-468 cells.

To investigate the molecular mechanisms underlying the biological functions of CDCA4, we performed RNA-sequencing (RNA-seq) analysis in TNBC cells following CDCA4 knockdown. Differential expression analysis revealed that 106 genes were upregulated and 673 genes were downregulated upon CDCA4 silencing (Figure 3A). Hierarchical clustering analysis showed a clear separation between the control and CDCA4-knockdown groups, with high consistency among samples within each group (Figure 3B).

To further explore the downstream pathways regulated by CDCA4, KEGG pathway enrichment analysis was performed using the differentially expressed genes. The enriched pathways included lipid metabolism-related pathways as well as metabolism-associated signaling pathways, such as mTOR signaling, suggesting that CDCA4 may be involved in the regulation of metabolic processes in TNBC cells (Figure 3C).

To validate the transcriptomic findings, we next examined lipid metabolism-associated parameters in TNBC cells after CDCA4 knockdown. In MDA-MB-468 cells, CDCA4 silencing significantly reduced the intracellular levels of free fatty acids (FFA), total cholesterol (TC), and triglycerides (TG), and Oil Red O staining showed a marked decrease in intracellular lipid accumulation (Figure 3D–G). Similar results were observed in HCC1937 cells following CDCA4 knockdown (Figure S2A–D). Furthermore, Western blot analysis demonstrated that the protein levels of the key lipogenic enzymes fatty acid synthase (FASN) and acetyl-CoA carboxylase 1 (ACC1) were markedly decreased after CDCA4 depletion in both MDA-MB-468 and HCC1937 cells (Figure 3H and Figure S2E).

To further determine whether CDCA4 knockdown affects other lipid metabolism-related processes, we examined the expression of SCD1, ACLY, and CPT1A, as well as oxygen consumption rate (OCR). RT-qPCR analysis showed that the mRNA levels of SCD1, ACLY, and CPT1A were not significantly changed after CDCA4 knockdown (Figure S2F). Consistently, these genes did not show significant alterations in the RNA-seq dataset. In addition, OCR analysis revealed no significant difference among the control and CDCA4-knockdown groups (Figure S2G), suggesting that CDCA4 depletion does not markedly affect fatty acid oxidation under the current experimental conditions. Collectively, these findings indicate that CDCA4 contributes to lipid metabolic reprogramming in TNBC cells, primarily by regulating intracellular lipid accumulation and lipogenic enzyme expression rather than by significantly altering fatty acid oxidation.

### 3.4. Rescue of CDCA4 Expression Reverses the Effects of Lipid Metabolic Reprogramming

To confirm the specificity of CDCA4 knockdown-induced effects, we performed rescue experiments in MDA-MB-468 cells by re-expressing CDCA4. RT-qPCR confirmed efficient CDCA4 knockdown and restoration after CDCA4 re-expression (Figure S3A). Western blot analysis showed that CDCA4 knockdown reduced p-mTOR levels, whereas CDCA4 re-expression restored p-mTOR expression (Figure S3B).

Functionally, CDCA4 re-expression partially rescued the inhibitory effect of CDCA4 knockdown on cell viability (Figure S3C). Moreover, the reductions in intracellular FFA, TC, and TG levels caused by CDCA4 depletion were also partially reversed by CDCA4 re-expression (Figure S3D). These results support the specific role of CDCA4 in regulating mTOR pathway activity, TNBC cell growth, and lipid metabolism.

### 3.5. CDCA4 Regulates Lipid Metabolism Through the mTOR/SREBP1 Axis in TNBC Cells

**Figure 4:**
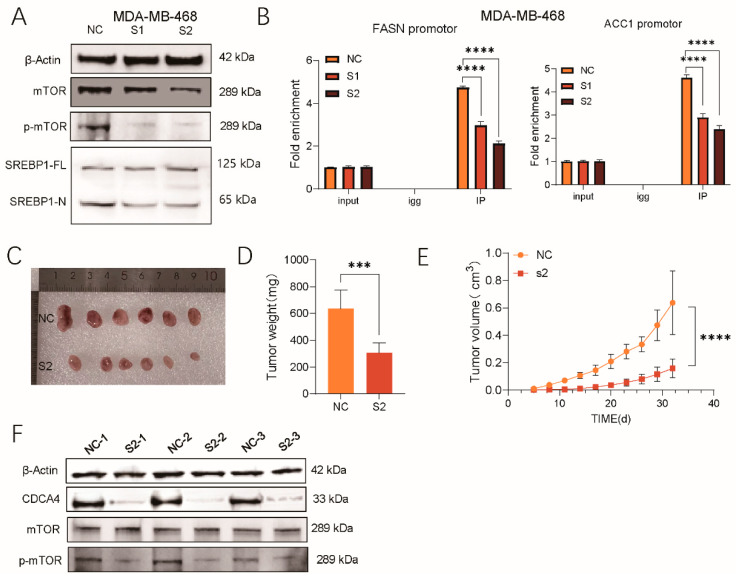
CDCA4 knockdown affects mTOR/SREBP1 signaling and tumor growth in MDA-MB-468 cells. (**A**) Western blot analysis of mTOR pathway activation and SREBP1-N expression in MDA-MB-468 cells following CDCA4 knockdown. (**B**) ChIP-qPCR analysis of SREBP1-N occupancy at the FASN and ACC1 promoter regions in MDA-MB-468 cells following CDCA4 knockdown. (**C**) Representative images of xenograft tumors formed by control and CDCA4-knockdown MDA-MB-468 cells. (**D**) Quantification of tumor weights in the xenograft model. (**E**) Tumor growth curves of xenografts derived from control and CDCA4-knockdown MDA-MB-468 cells. (**F**) Western blot analysis of proteins in xenograft tumor tissues.

To further investigate the mechanism by which CDCA4 regulates lipid metabolism, we integrated the published evidence with our RNA-seq data and hypothesized that CDCA4 might promote lipid metabolic reprogramming by activating the mTOR pathway and facilitating SREBP1 nuclear activation [23,25]. To test this hypothesis, we first examined mTOR signaling in TNBC cells following CDCA4 knockdown. Western blot analysis showed that CDCA4 silencing markedly reduced the phosphorylation level of mTOR in both MDA-MB-468 and HCC1937 cells. Meanwhile, the level of the active N-terminal form of SREBP1 (SREBP1-N) was also significantly decreased after CDCA4 depletion (Figure 4A and Figure S4A). Furthermore, ChIP-qPCR analysis demonstrated that CDCA4 knockdown reduced the binding of SREBP1-N to the promoter regions of the lipogenic genes FASN and ACC1 in both TNBC cell lines (Figure 4B and Figure S4B). These results indicate that CDCA4 may regulate lipid metabolism in TNBC cells, at least in part, through activation of the mTOR/SREBP1 signaling axis.

To further validate the oncogenic role of CDCA4 in vivo, a xenograft tumor model was established using MDA-MB-468 cells with stable CDCA4 knockdown. Compared with the control group, tumors derived from CDCA4-silenced cells were markedly smaller in size (Figure 4C). Consistently, tumor weight was significantly reduced in the CDCA4-knockdown group (Figure 4D), and tumor growth was substantially slowed throughout the experimental period (Figure 4E). In addition, Western blot analysis of xenograft tumor tissues showed that CDCA4 knockdown was accompanied by inhibition of mTOR pathway activation in vivo (Figure 4F). Collectively, these findings support a role for CDCA4 in promoting TNBC progression and lipid metabolic regulation through the mTOR/SREBP1 axis.

### 3.6. Inhibition of mTOR Reverses CDCA4-Induced Malignant Phenotypes and Lipid Metabolic Reprogramming in TNBC Cells

**Figure 5:**
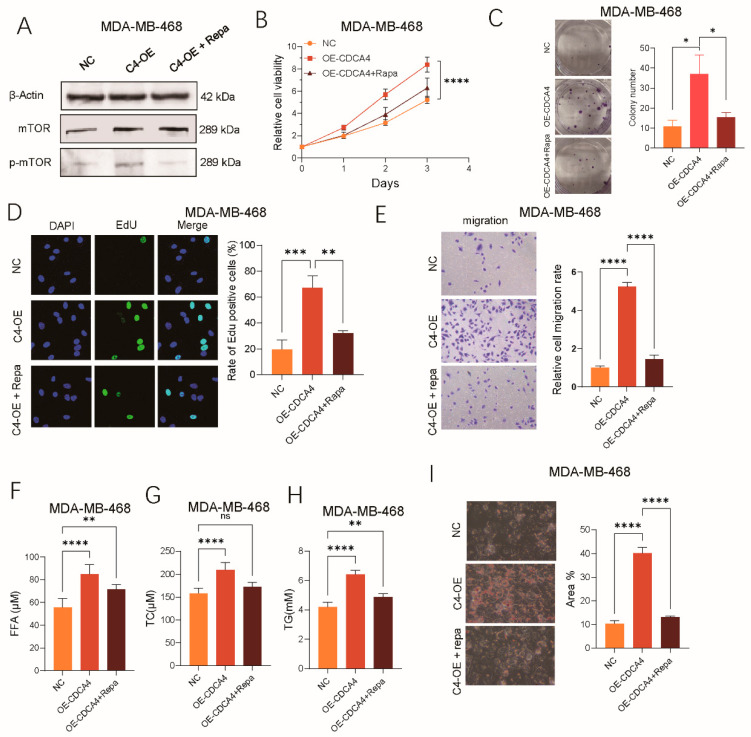
Rescue experiments in CDCA4-overexpressing TNBC cells treated with an mTOR pathway inhibitor. (**A**) Western blot analysis of mTOR pathway activation in CDCA4-overexpressing MDA-MB-468 cells treated with an mTOR inhibitor. (**B**) MTS assay in CDCA4-overexpressing MDA-MB-468 cells with or without mTOR inhibitor treatment. (**C**) EdU incorporation assay in CDCA4-overexpressing MDA-MB-468 cells with or without mTOR inhibitor treatment. (**D**) Colony formation assay in CDCA4-overexpressing MDA-MB-468 cells with or without mTOR inhibitor treatment. (**E**) Transwell migration and invasion assays in CDCA4-overexpressing MDA-MB-468 cells with or without mTOR inhibitor treatment. (**F**–**I**) Quantification of intracellular free fatty acid (FFA) levels (**F**), total cholesterol (TC) content (**G**), triglyceride (TG) content (**H**), and Oil Red O staining (**I**) in CDCA4-overexpressing MDA-MB-468 cells with or without mTOR inhibitor treatment.

To further determine whether CDCA4 regulates lipid metabolism through the mTOR pathway in TNBC cells, rescue experiments were performed by treating CDCA4-overexpressing cells with an mTOR pathway inhibitor. The inhibitory effect on mTOR signaling was first confirmed by Western blot analysis (Figure 5A). Functional assays showed that pharmacological inhibition of mTOR markedly attenuated the CDCA4 overexpression-induced malignant phenotypes. Specifically, MTS, EdU and colony formation assays demonstrated that the enhanced proliferative capacity caused by CDCA4 overexpression was significantly impaired following mTOR inhibition (Figure 5B–D). In addition, Transwell assays showed that the increased migratory abilities induced by CDCA4 overexpression were substantially reversed by mTOR pathway blockade (Figure 5E).

Given the involvement of CDCA4 in lipid metabolic regulation, we further examined whether mTOR inhibition could counteract CDCA4-induced lipid metabolic alterations. Biochemical analyses showed that the elevated intracellular levels of free fatty acids (FFA), total cholesterol (TC), and triglycerides (TG) in CDCA4-overexpressing cells were significantly reduced after treatment with the mTOR inhibitor (Figure 5F–H). Consistently, Oil Red O staining showed that mTOR inhibition markedly decreased the intracellular lipid accumulation induced by CDCA4 overexpression (Figure 5I). Together, these findings indicate that CDCA4 promotes malignant progression and lipid metabolic reprogramming in TNBC cells, at least in part, through activation of the mTOR pathway.

### 3.7. CDCA4 Activates mTOR Signaling and Malignant Phenotypes in TNBC Cells Through SESN2

**Figure 6:**
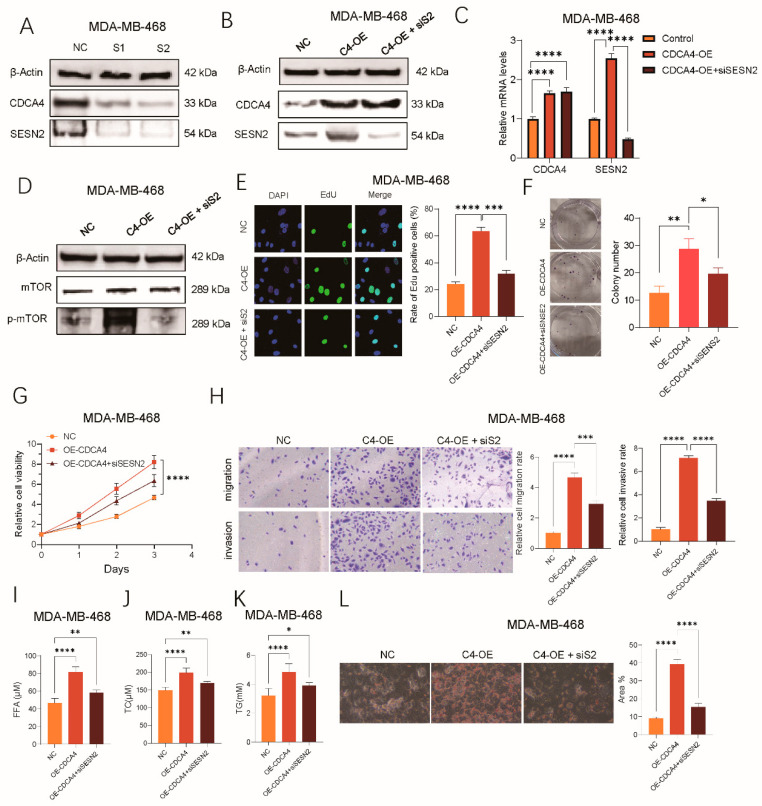
Rescue experiments examining the role of SESN2 in CDCA4-mediated mTOR activation and malignant phenotypes in TNBC cells. (**A**) Western blot analysis of SESN2 expression in TNBC cells following CDCA4 knockdown. (**B**) Western blot analysis of CDCA4 and SESN2 expression in TNBC cells with CDCA4 overexpression and SESN2 knockdown. (**C**) RT-qPCR analysis of CDCA4 and SESN2 expression in TNBC cells with CDCA4 overexpression and SESN2 knockdown. (**D**) Western blot analysis of mTOR pathway activation in TNBC cells with CDCA4 overexpression and SESN2 knockdown. (**E**) EdU incorporation assay in TNBC cells with CDCA4 overexpression and SESN2 knockdown. (**F**) Colony formation assay in TNBC cells with CDCA4 overexpression and SESN2 knockdown. (**G**) MTS assay in TNBC cells with CDCA4 overexpression and SESN2 knockdown. (**H**) Transwell migration assay in TNBC cells with CDCA4 overexpression and SESN2 knockdown. (**I**–**L**) Quantification of intracellular free fatty acid (FFA) levels (**I**), total cholesterol (TC) content (**J**), triglyceride (TG) content (**K**), and Oil Red O staining (**L**) in CDCA4-overexpressing MDA-MB-468 cells with or without SESN2 knockdown.

To further clarify the mechanism by which CDCA4 activates mTOR signaling, we sought to identify upstream regulators of mTOR that are modulated by CDCA4. Western blot analysis showed that SESN2 expression was markedly decreased following CDCA4 knockdown in TNBC cells (Figure 6A). Based on this finding and previous reports implicating SESN2 in the regulation of mTOR signaling, we next investigated whether the CDCA4/SESN2 axis contributes to mTOR activation and the malignant phenotype of TNBC cells.

To this end, rescue experiments were performed in TNBC cells by overexpressing CDCA4 in combination with SESN2 knockdown. Western blot and RT-qPCR analyses showed that CDCA4 overexpression increased SESN2 expression, whereas silencing SESN2 did not affect CDCA4 expression, supporting that SESN2 acts downstream of CDCA4 (Figure 6B,C). In addition, Western blot analysis demonstrated that SESN2 knockdown attenuated mTOR pathway activation induced by CDCA4 overexpression (Figure 6D). Functional assays further showed that depletion of SESN2 significantly reversed the enhancement of cell proliferation and migration caused by CDCA4 overexpression, as evidenced by EdU, MTS, colony formation and Transwell assays (Figure 6E–H). We also performed reciprocal rescue experiments by re-expressing SESN2 in CDCA4-knockdown MDA-MB-468 cells. RT-qPCR analysis confirmed that SESN2 expression was effectively restored, whereas CDCA4 expression remained suppressed (Figure S5A). Western blot analysis showed that SESN2 overexpression partially restored p-mTOR levels reduced by CDCA4 knockdown (Figure S5B). Functionally, SESN2 re-expression partially rescued the inhibitory effect of CDCA4 depletion on cell viability (Figure S5C). In addition, SESN2 overexpression partially reversed the reductions in intracellular FFA, TC, and TG levels induced by CDCA4 knockdown (Figure S5D). Collectively, these results suggest that CDCA4 promotes mTOR pathway activation and malignant progression in TNBC cells, at least in part, through SESN2.

**Figure 7:**
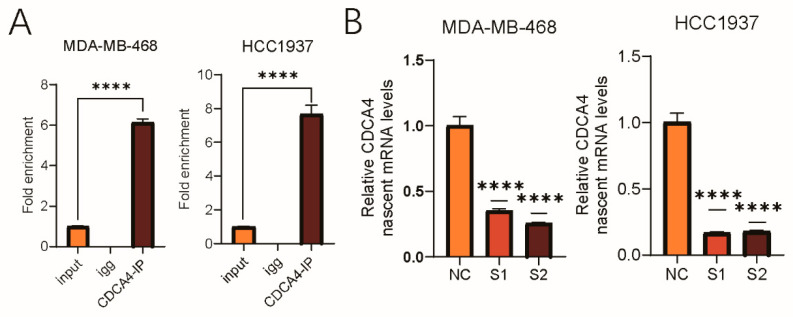
CDCA4 promotes SESN2 transcription in TNBC cells. (**A**) ChIP-qPCR analysis showing the enrichment of CDCA4 at the SESN2 promoter region in MDA-MB-468 and HCC1937 cells. IgG was used as a negative control. (**B**) 4s-U labeling followed by nascent mRNA pull-down analysis of newly synthesized SESN2 mRNA levels in control and CDCA4-knockdown MDA-MB-468 and HCC1937 cells.

To further investigate whether CDCA4 regulates SESN2 at the transcriptional level, we performed ChIP-qPCR analysis to assess the enrichment of CDCA4 at the SESN2 promoter region (Figure 7A). Furthermore, we performed 4s-U labeling followed by nascent mRNA pull-down. CDCA4 knockdown significantly reduced newly synthesized SESN2 mRNA levels in both MDA-MB-468 and HCC1937 cells (Figure 7B). These findings indicate that CDCA4 contributes to the transcriptional regulation of SESN2, thereby providing mechanistic support for SESN2 as a downstream mediator of CDCA4 in the regulation of mTOR/SREBP1 signaling in TNBC cells.

## 4. Discussion

In the present study, we identified CDCA4 as a previously underappreciated oncogenic driver in triple-negative breast cancer (TNBC) through integrative analysis of public datasets and functional validation in vitro and in vivo. We found that CDCA4 was significantly upregulated in breast cancer tissues, particularly in TNBC, and that elevated CDCA4 expression was associated with poor overall survival in TNBC patients. Functional assays further demonstrated that CDCA4 promoted TNBC cell proliferation, migration, invasion, and xenograft tumor growth. These findings support a tumor-promoting role of CDCA4 in TNBC. CDCA4 has been characterized as a cell cycle-associated nuclear protein involved in transcriptional regulation and cell proliferation, and accumulating evidence suggests that it exerts protumorigenic functions in multiple malignancies [7,9]. In breast cancer, Pang et al. reported that CDCA4 knockdown inhibited the proliferation of MDA-MB-231 TNBC cells in vitro and suppressed xenograft tumor growth in vivo [9]. In addition, Zhang et al. showed that the STAT1/circIFI30/CDCA4 axis promoted TNBC cell proliferation, migration, invasion, and EMT-related phenotypes [13]. Our findings are consistent with these previous reports and further support the oncogenic role of CDCA4 in TNBC. Importantly, while earlier studies mainly focused on the role of CDCA4 in proliferation, apoptosis, and EMT-related phenotypes, our study extends the biological significance of CDCA4 by linking it to lipid metabolic reprogramming and mTOR/SREBP1-dependent lipogenic regulation.

Metabolic plasticity is increasingly recognized as a critical determinant of TNBC aggressiveness [3,26]. Among different metabolic alterations, dysregulated lipid metabolism has emerged as an important feature that supports rapid tumor growth, membrane biosynthesis, energy storage, redox balance, metastatic potential, and therapeutic resistance [17]. TNBC cells exhibit high metabolic demand and frequently rely on enhanced lipogenesis to support membrane biosynthesis, energy storage, redox balance, and metastatic behavior [6]. Several lipid metabolism regulators, including SREBP1, FASN, ACC1, SCD1, and upstream epigenetic or signaling regulators, have been implicated in TNBC growth and lipid biosynthesis. For example, recent studies have shown that activation of the FLAD1/LSD1/SREBP1 axis promotes lipogenic enzyme expression and TNBC progression, whereas targeting lipid metabolic regulators suppresses TNBC growth [27]. These observations provide an important biological context for our finding that CDCA4 regulates lipid accumulation and lipogenic enzyme expression in TNBC cells.

Among the signaling pathways involved in this process, the mTOR pathway is a key regulator linking oncogenic signaling to anabolic metabolism [20,28]. Previous studies have shown that aberrant mTOR activation promotes tumor cell proliferation and survival while also enhancing lipogenesis through activation of SREBP1, which in turn drives the transcription of key lipogenic enzymes such as FASN and ACC1 [23,29,30]. In the current study, RNA-seq analysis of CDCA4-silenced cells revealed enrichment of lipid metabolism-related pathways and mTOR-associated signaling. Consistently, CDCA4 knockdown reduced intracellular levels of FFA, TC, and TG, decreased lipid accumulation, and downregulated FASN and ACC1 expression. Additional analyses showed that fatty acid oxidation-related genes, including SCD1, ACLY, and CPT1A, were not significantly altered after CDCA4 knockdown, and OCR analysis showed no significant change in mitochondrial oxygen consumption. Therefore, under the present experimental conditions, CDCA4 appears to mainly affect lipid accumulation and lipogenic enzyme expression rather than broadly altering fatty acid oxidation or mitochondrial respiration.

A key issue raised by our findings is how CDCA4 regulates mTOR/SREBP1 signaling. We identified SESN2 as a downstream mediator linking CDCA4 to mTOR pathway activation. We found that CDCA4 knockdown reduced SESN2 expression at both the mRNA and protein levels, whereas SESN2 knockdown did not affect CDCA4 expression, suggesting that SESN2 functions downstream of CDCA4. However, the role of SESN2 in mTOR signaling requires careful interpretation. SESN2 is classically regarded as a stress-inducible suppressor of mTORC1, particularly under nutrient stress or amino acid deprivation conditions, through mechanisms involving GATOR complex regulation and AMPK activation [31,32]. By contrast, accumulating evidence suggests that SESN2 biology is context-dependent. In addition to its inhibitory role toward mTORC1, SESN2 has been reported to participate in AKT/mTORC2-associated signaling under specific stress or tumor contexts [33,34,35]. Other studies also suggest that SESN2 may have dual roles in cancer biology depending on tumor type, metabolic state and cellular stress [33]. Therefore, SESN2 should not necessarily be viewed as a uniformly inhibitory regulator of the entire mTOR network [36,37]. In this context, our data suggest that, in TNBC cells, CDCA4-induced SESN2 expression may support a pro-tumorigenic mTOR/SREBP1 signaling state that promotes lipogenic reprogramming. Nevertheless, we acknowledge that our current data do not fully define the precise complex-specific mechanism by which SESN2 regulates mTOR signaling. The present study primarily evaluated overall mTOR pathway activity and downstream SREBP1-mediated lipogenic outputs, rather than systematically distinguishing mTORC1-specific from mTORC2-specific regulation. Therefore, although our findings support a CDCA4/SESN2/mTOR/SREBP1 regulatory model, further studies are required to determine how SESN2 coordinates mTORC1 and mTORC2 activity in TNBC cells.

Several limitations should be acknowledged. First, although ChIP and 4s-U nascent mRNA analyses support a role for CDCA4 in SESN2 transcriptional regulation, additional studies, such as SESN2 promoter luciferase reporter assays, promoter truncation analysis, and identification of CDCA4-associated transcriptional cofactors, are needed to fully define the transcriptional mechanism. Second, the precise mTOR complex involved in SESN2-mediated signaling remains unresolved. Future studies using genetic or pharmacological interrogation of mTORC1- and mTORC2-specific components, such as RAPTOR and RICTOR, will be necessary. Third, although our in vitro and in vivo data support a tumor-promoting role of CDCA4, the clinical relevance of the CDCA4/SESN2/mTOR/SREBP1 axis requires validation in larger independent TNBC cohorts and well-characterized clinical specimens. Finally, TNBC is a heterogeneous disease, and additional TNBC models will be needed to determine whether this regulatory mechanism is broadly applicable across different molecular subtypes.

In summary, our study demonstrates that CDCA4 is highly expressed in TNBC and functions as an oncogenic factor that promotes tumor progression and lipid metabolic reprogramming. Mechanistically, our data support a regulatory model in which CDCA4 enhances SESN2 expression and activates mTOR/SREBP1-associated lipogenic transcription, thereby contributing to malignant phenotypes in TNBC. These findings broaden the current understanding of CDCA4 biology and reveal a potential mechanistic link between a cell cycle-associated regulator and lipid metabolic rewiring in TNBC. Targeting this CDCA4-centered signaling network may represent a potential therapeutic strategy, although further mechanistic and clinical validation is warranted.

## Data Availability

The RNA-seq datasets generated and analyzed in the present study have been deposited in the NCBI Gene Expression Omnibus (GEO) database under accession number GSE330488. All other data supporting the findings of this study are available from the corresponding authors upon reasonable request.

## Supplementary Materials

The following supporting information can be downloaded at: https://www.mdpi.com/article/10.3390/cancers18152517/s1, Figure S1: Functional analyses of CDCA4 knockdown in HCC1937 cells; Figure S2. Lipid metabolic analyses in HCC1937 cells after CDCA4 knockdown; Figure S3. Rescue of CDCA4 expression reverses the effects of CDCA4 knockdown; Figure S4. CDCA4 knockdown affects mTOR/SREBP1 signaling in HCC1937 cells; Figure S5. SESN2 overexpression partially rescues the effects of CDCA4 knockdown in MDA-MB-468 cells; Table S1: Sequences of oligonucleotides used in this study; Table S2: Supplementary File S1. Supplementary File S2: Unedited original image of western blot.

## Abbreviations

TCGA	The Cancer Genome Atlas
ER	estrogen receptor
HER2	human epidermal growth factor receptor 2
MTS	3-(45-dimethylthiazol-2-yl)-5-(3-carboxymethoxyphenyl)-2-(4-sulfophenyl)-2H-tetrazolium
OS	overall survival
PARP	poly(ADP-ribose) polymerase
PI3K	phosphoinositide 3-kinase
PR	progesterone receptor
RNA-seq	RNA sequencing
DEGs	differentially expressed genes
KEGG	Kyoto Encyclopedia of Genes and Genomes
RT-qPCR	reverse transcription quantitative real-time PCR
CDCA4	cell division cycle-associated 4
ACC1	acetyl-CoA carboxylase 1
FASN	fatty acid synthase
SESN2	sestrin 2
SREBP1	sterol regulatory element-binding protein 1
SREBP1-N	N-terminal active fragment of SREBP1
TC	total cholesterol
TG	triglyceride
FFA	free fatty acid
TNBC	triple-negative breast cancer
