# Supplementary material for: CDCA4 Promotes Lipid Metabolism in Triple-Negative Breast Cancer Through Activation of the SESN2/mTOR/SREBP1 Pathway
Source: Cancers (Basel). 2026 Aug 6;18(15):2517. doi: 10.3390/cancers18152517 (PMC13465203; doi:10.3390/cancers18152517)

This experiment used three types of markers.

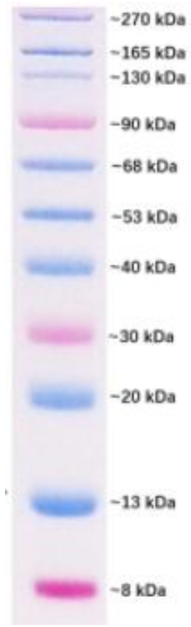

Prestained Protein Marker Ⅷ (8-270 kDa)

Product Number : G2089-250UL

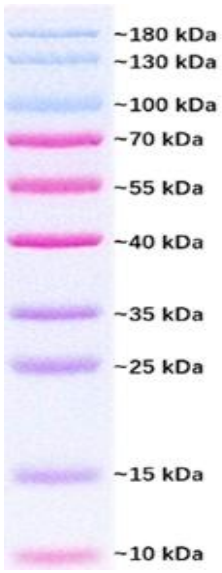

Prestained ProteinMarker Ⅹ (10-180 kDa)

Product Number : G2091-250UL

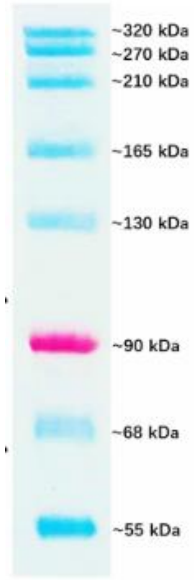

Prestained Protein Marker Ⅵ (55-320 kDa)

Product Number : G2085-250UL

The antibodies used

	Antibody Name	Brand	Molecular weight	Product Number
1	mTOR Monoclonal antibody	proteintech	289 kDa	66888-1-Ig
2	Phospho-mTOR (Ser2448) Monoclonal antibody	proteintech	289 kDa	67778-1-Ig
3	SREBP1 antibody	Abmart	68, 125kDa	PU288919S
4	Sestrin 2 Polyclonal antibody	proteintech	54-60 kDa	10795-1-AP
5	CDCA4 Polyclonal antibody	proteintech	33 kDa	11625-1-AP
6	ACC1 Polyclonal antibody	proteintech	250kDa	21923-1-AP
7	FASN Polyclonal antibody	proteintech	250kDa	10624-2-AP-50ul
8	ABclonal HRP-conjugated Goat anti-Mouse IgG (H+L)	Abclonal		AS003
9	ABclonal HRP-conjugated Goat anti-Rabbit IgG (H+L)	Abclonal		AS014
10	Beta Actin Recombinant antibody	proteintech	42 kDa	81115-1-RR

Figure 3H.

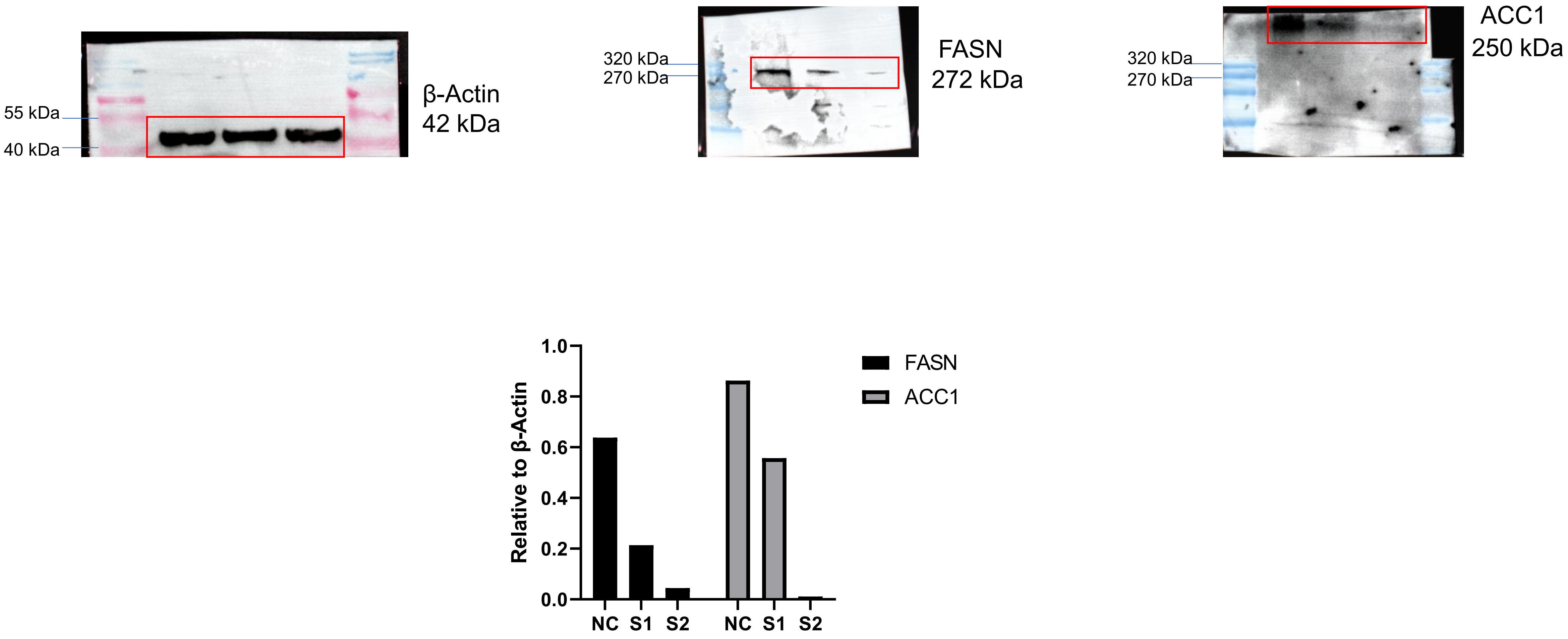
55 kDa
40 kDa
β-Actin
42 kDa
320 kDa
270 kDa
FASN
272 kDa
320 kDa
270 kDa
ACC1
250 kDa
Relative to β-Actin
1.0
0.8
0.6
0.4
0.2
0.0
NC
S1
S2
NC
S1
S2
FASN
ACC1

Figure 4A.

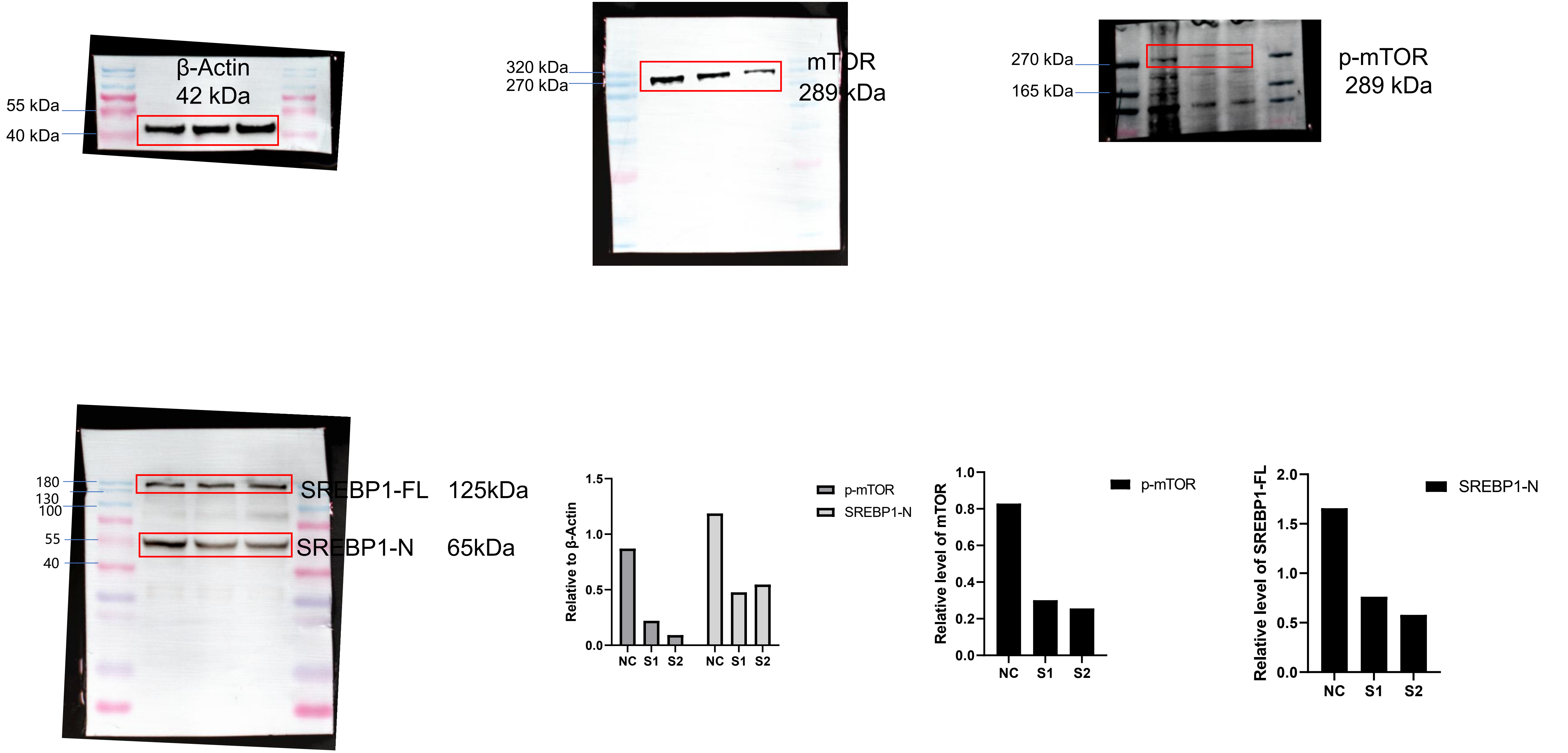
β-Actin
42 kDa
55 kDa
40 kDa
mTOR
289 kDa
320 kDa
270 kDa
p-mTOR
289 kDa
270 kDa
165 kDa
SREBP1-FL 125kDa
SREBP1-N 65kDa
180
130
100
55
40
Relative to β-Actin
p-mTOR
SREBP1-N
1.5
1.0
0.5
0.0
NC S1 S2 NC S1 S2
Relative level of mTOR
p-mTOR
1.0
0.8
0.6
0.4
0.2
0.0
NC S1 S2
Relative level of SREBP1-FL
SREBP1-N
2.0
1.5
1.0
0.5
0.0
NC S1 S2

Figure 4F.

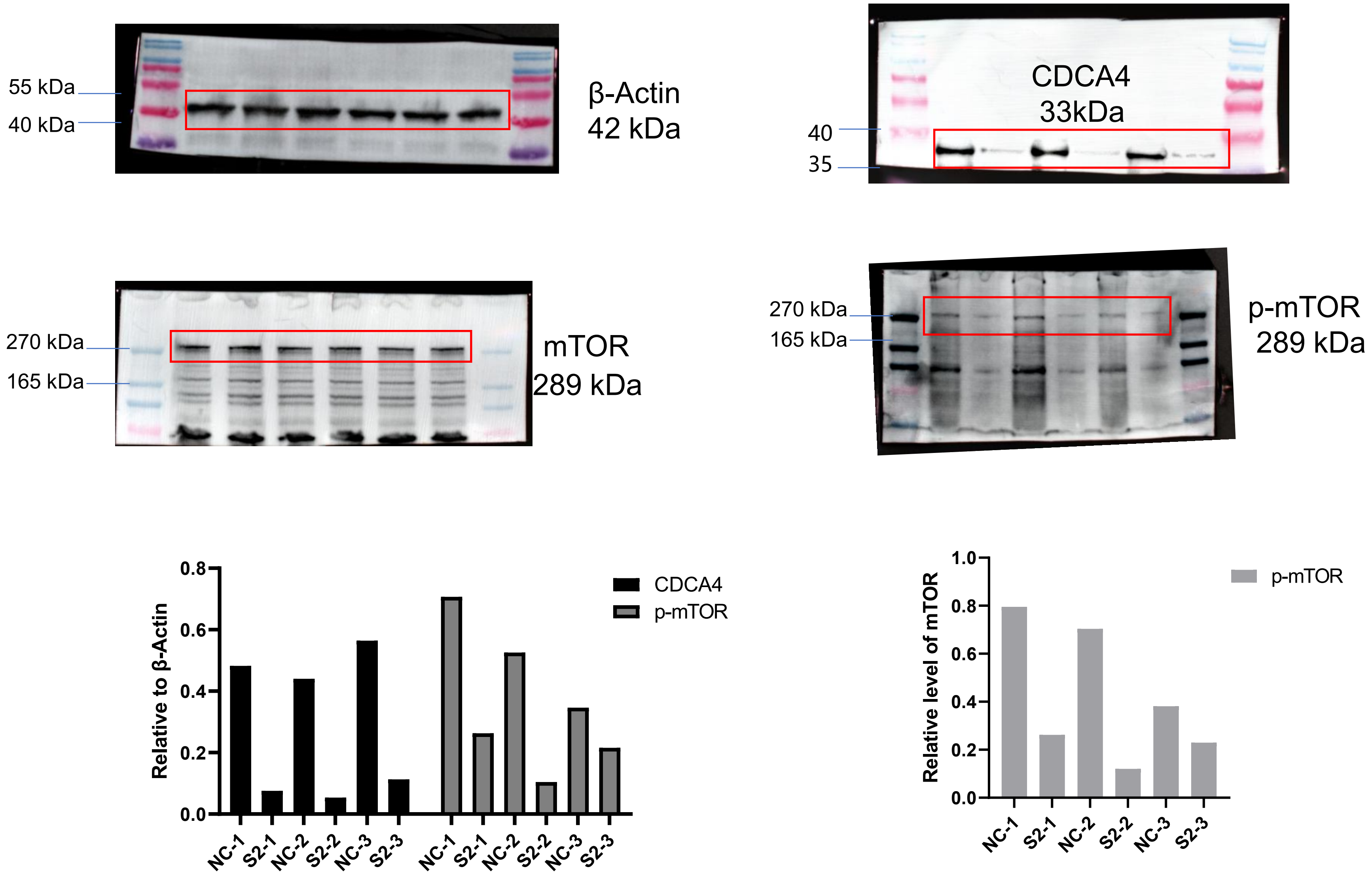
55 kDa
40 kDa
β-Actin
42 kDa
CDCA4
33kDa
40
35
270 kDa
165 kDa
mTOR
289 kDa
270 kDa
165 kDa
p-mTOR
289 kDa
Relative to β-Actin
0.8
0.6
0.4
0.2
0.0
CDCA4
p-mTOR
NC-1
S2-1
NC-2
S2-2
NC-3
S2-3
NC-1
S2-1
NC-2
S2-2
NC-3
S2-3
Relative level of mTOR
1.0
0.8
0.6
0.4
0.2
0.0
p-mTOR
NC-1
S2-1
NC-2
S2-2
NC-3
S2-3

Figure 5A.

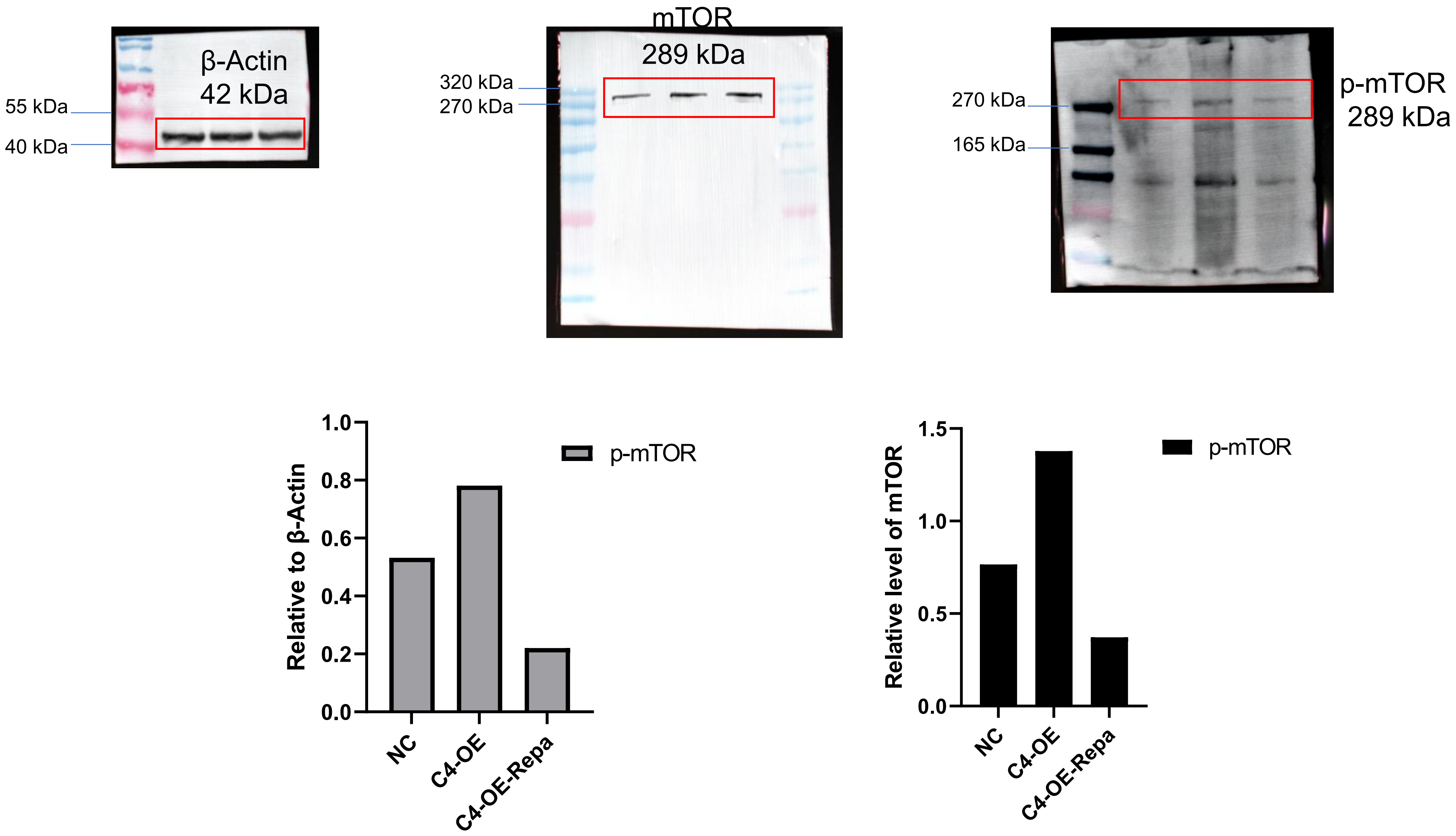

β-Actin
42 kDa
55 kDa
40 kDa
mTOR
289 kDa
320 kDa
270 kDa
p-mTOR
289 kDa
270 kDa
165 kDa
Relative to β-Actin
1.0
0.8
0.6
0.4
0.2
0.0
p-mTOR
NC
C4-OE
C4-OE-Repa
Relative level of mTOR
1.5
1.0
0.5
0.0
p-mTOR
NC
C4-OE
C4-OE-Repa

Figure 6A.

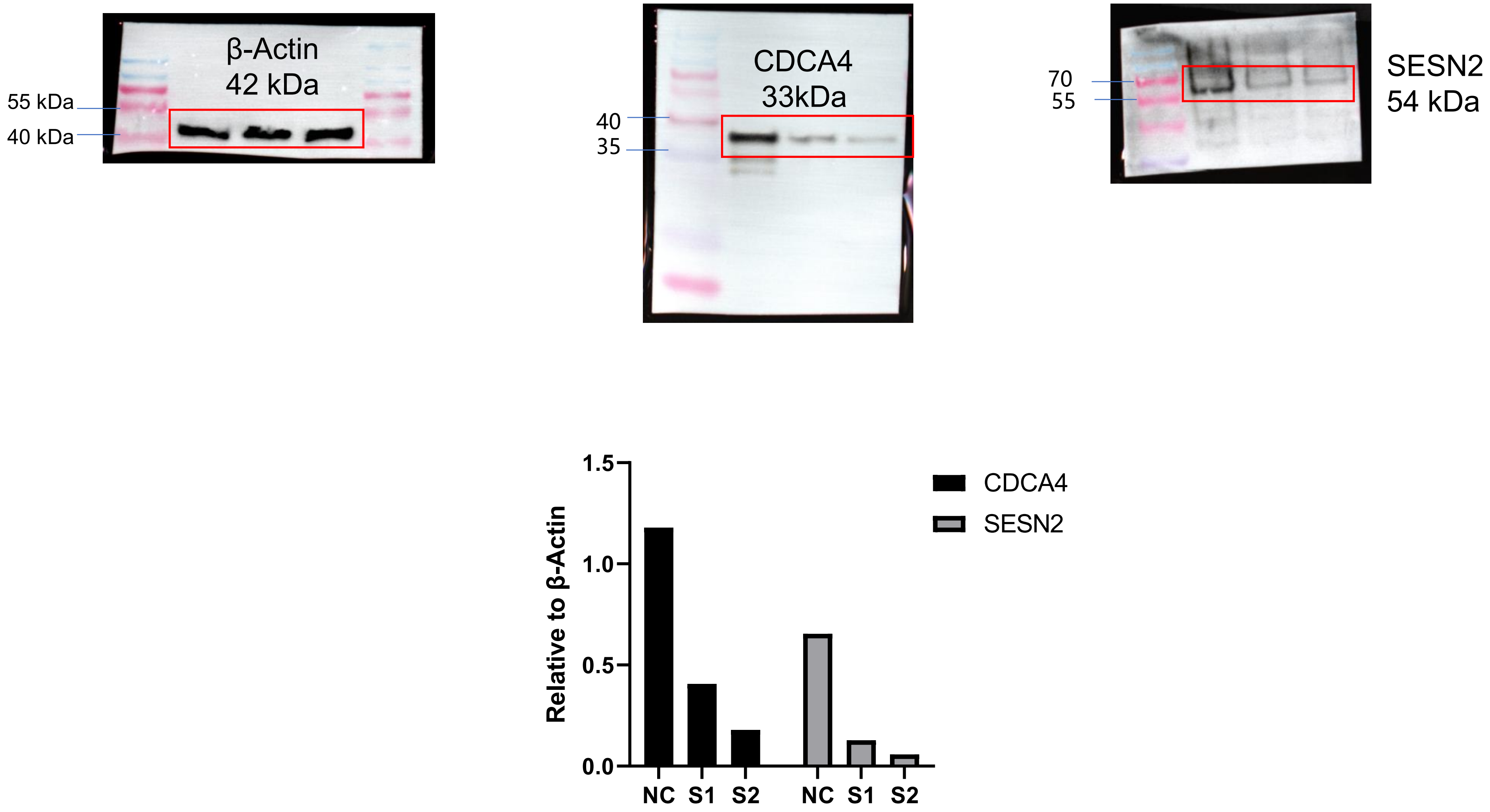

β-Actin
42 kDa
55 kDa
40 kDa
CDCA4
33kDa
40
35
70
55
SESN2
54 kDa
1.5
1.0
0.5
0.0
Relative to β-Actin
CDCA4
SESN2
NC
S1
S2
NC
S1
S2

Figure 6B.

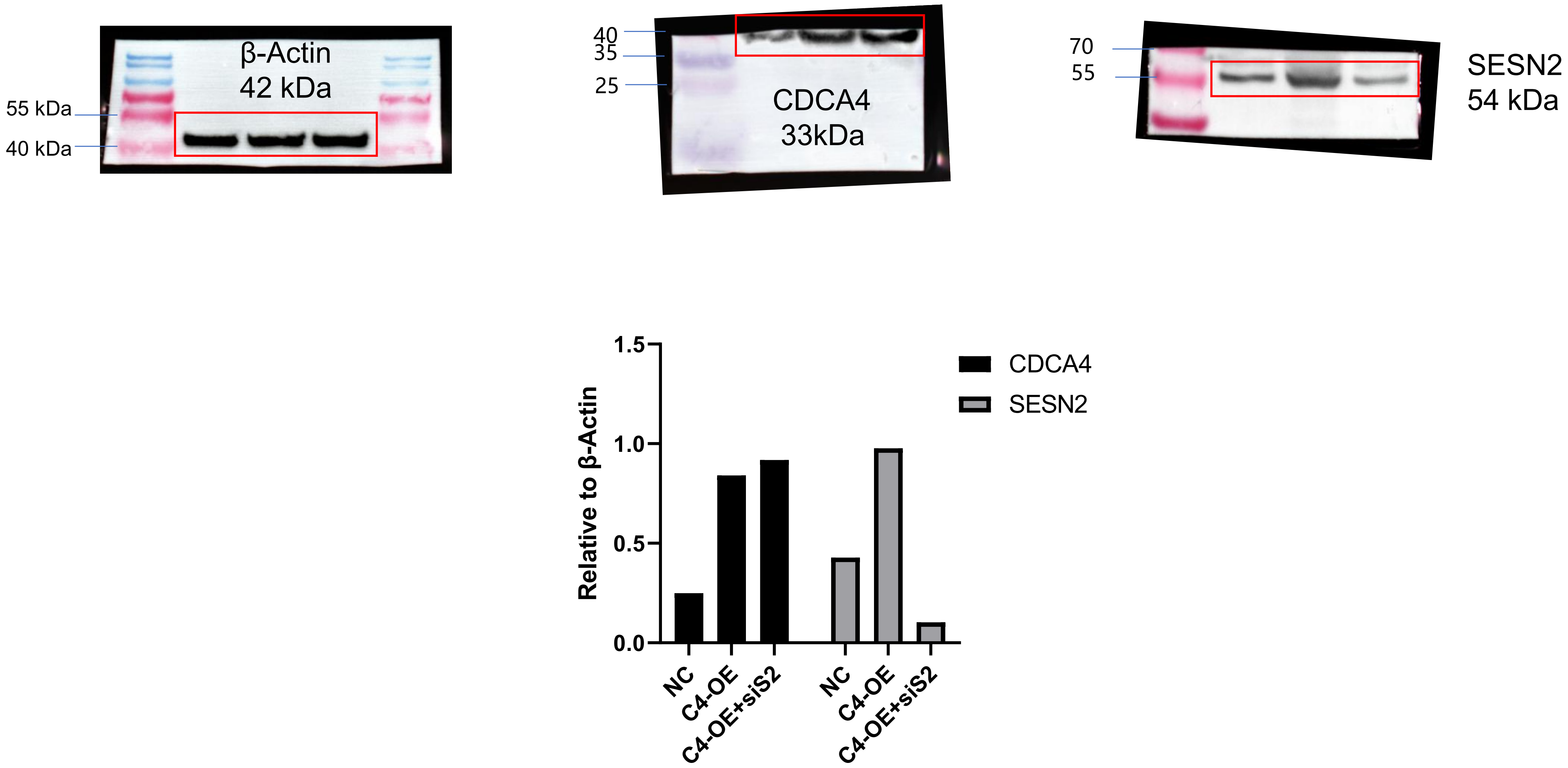

β-Actin
42 kDa
55 kDa
40 kDa
40
35
25
CDCA4
33kDa
70
55
SESN2
54 kDa
Relative to β-Actin
1.5
1.0
0.5
0.0
CDCA4
SESN2
NC
C4-OE
C4-OE+siS2
NC
C4-OE
C4-OE+siS2

Figure 6D.

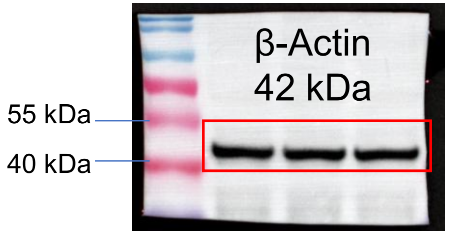

β-Actin
42 kDa
55 kDa
40 kDa

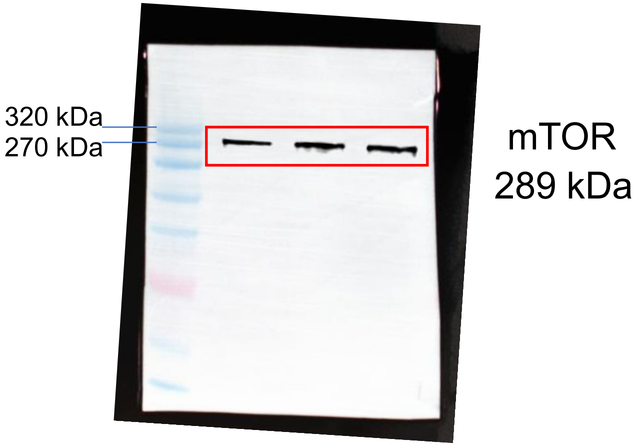

320 kDa
270 kDa
mTOR
289 kDa

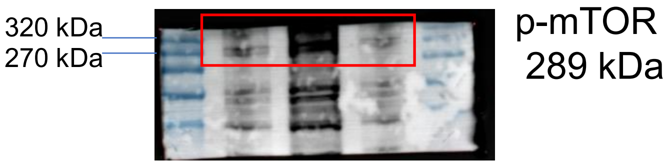

320 kDa
270 kDa
p-mTOR
289 kDa

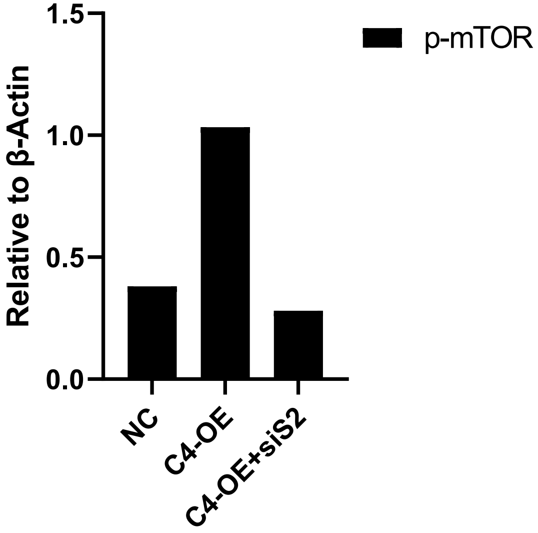

Relative to β-Actin
p-mTOR
0.0
0.5
1.0
1.5
NC
C4-OE
C4-OE+siS2

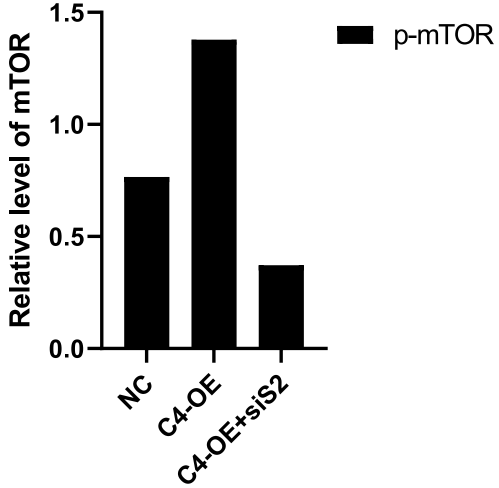

Relative level of mTOR
p-mTOR
0.0
0.5
1.0
1.5
NC
C4-OE
C4-OE+siS2

Figure S2E.

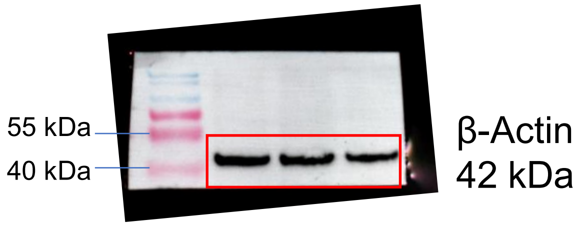
55 kDa
40 kDa
β-Actin
42 kDa

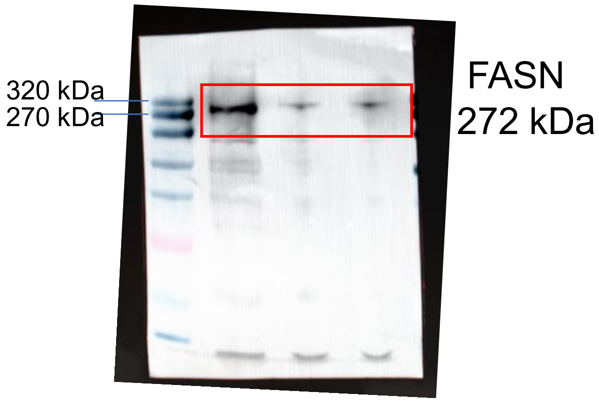
320 kDa
270 kDa
FASN
272 kDa

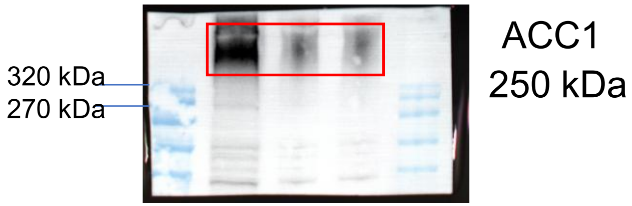
320 kDa
270 kDa
ACC1
250 kDa

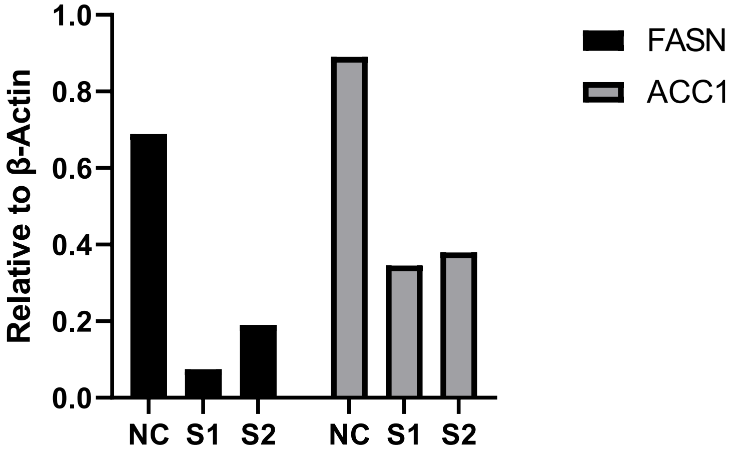
Relative to β-Actin
1.0
0.8
0.6
0.4
0.2
0.0
NC
S1
S2
NC
S1
S2
FASN
ACC1

Figure S3A.

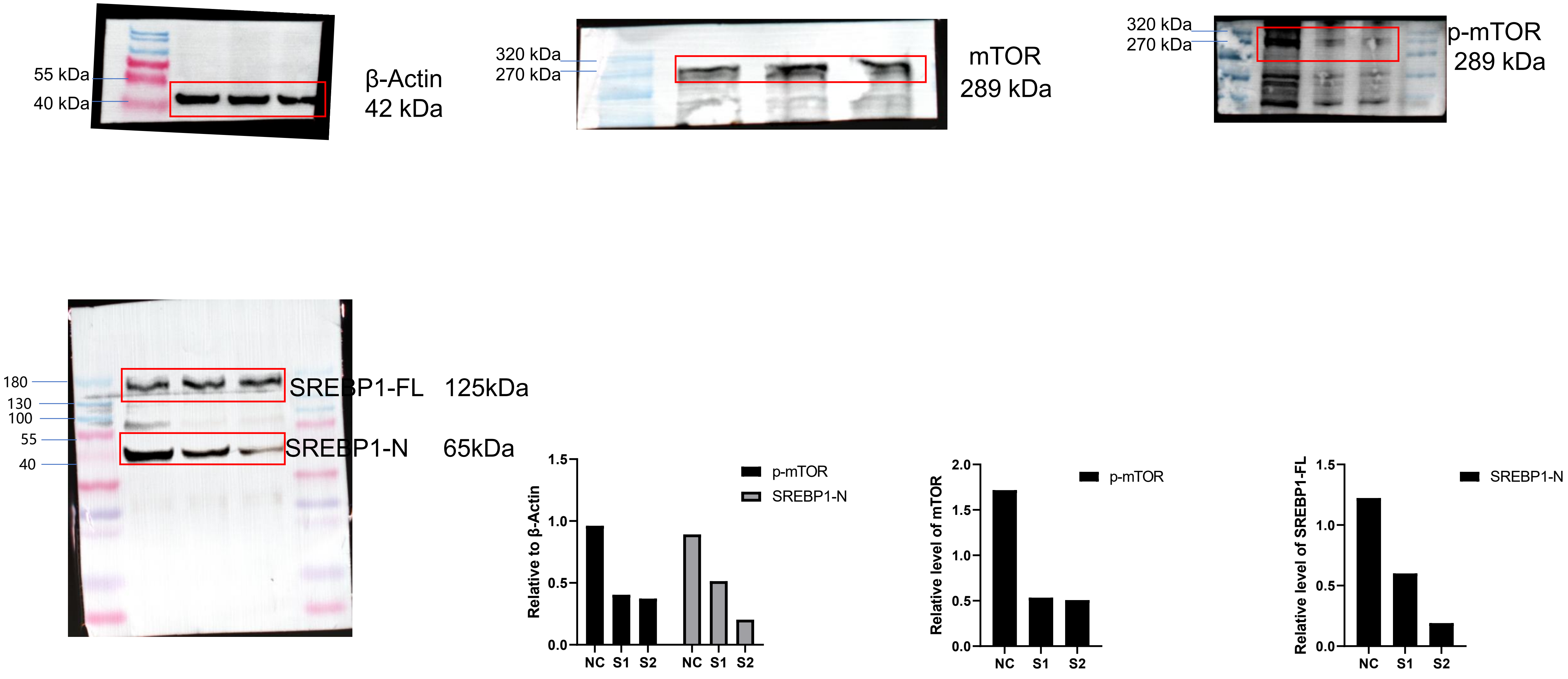

55 kDa
40 kDa
β-Actin
42 kDa
320 kDa
270 kDa
mTOR
289 kDa
320 kDa
270 kDa
p-mTOR
289 kDa
180
130
100
55
40
SREBP1-FL 125kDa
SREBP1-N 65kDa
Relative to β-Actin
p-mTOR
SREBP1-N
NC S1 S2 NC S1 S2
Relative level of mTOR
p-mTOR
NC S1 S2
Relative level of SREBP1-FL
SREBP1-N
NC S1 S2

New Supplementary Picture

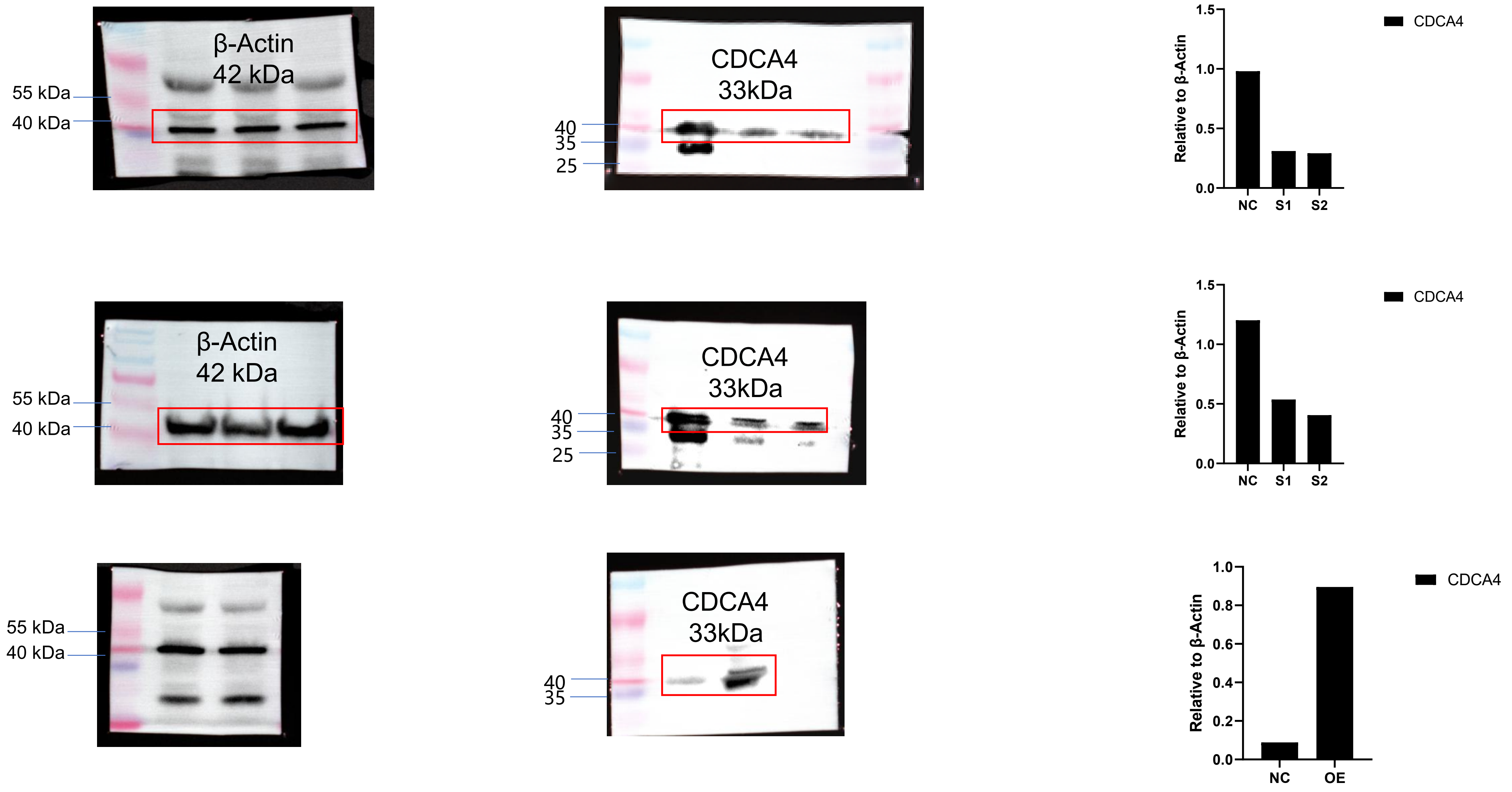

（NC， si-CDCA4， si-CDCA4 + CDCA4-rescue）

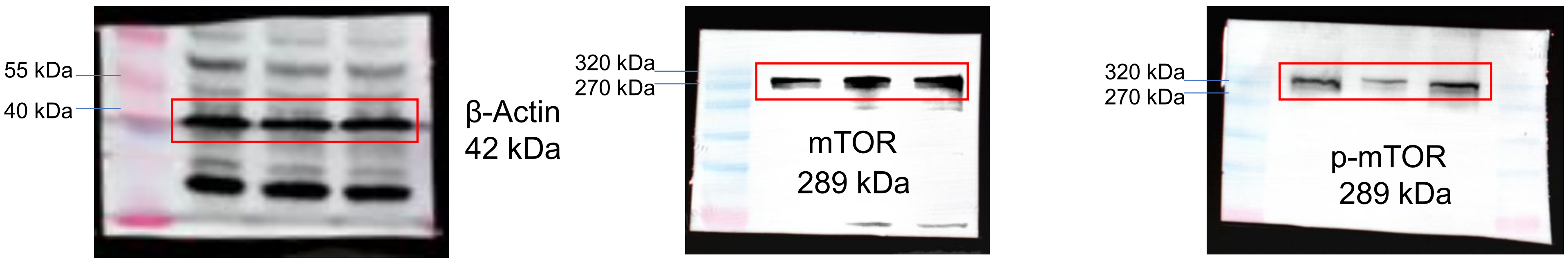

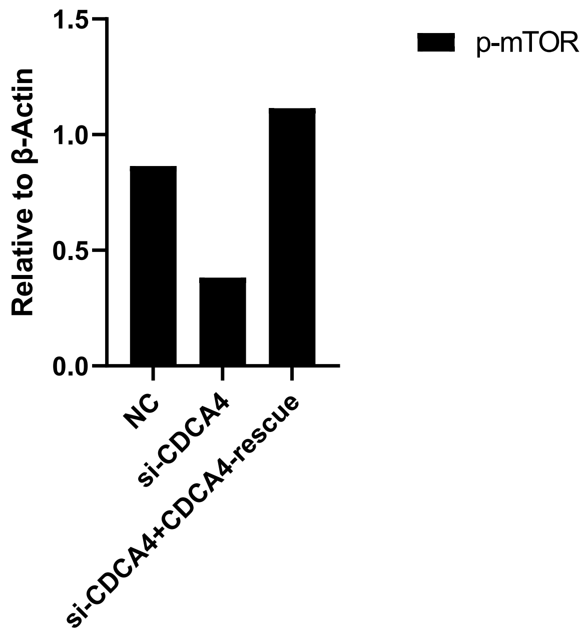

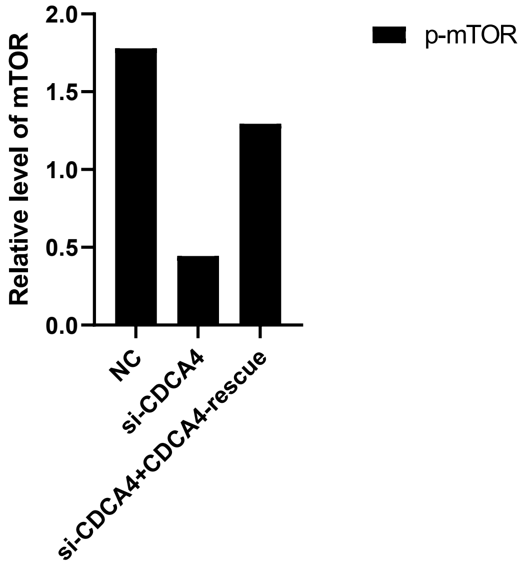

NC， si-CDCA4， si-CDCA4 + SESN2-OE

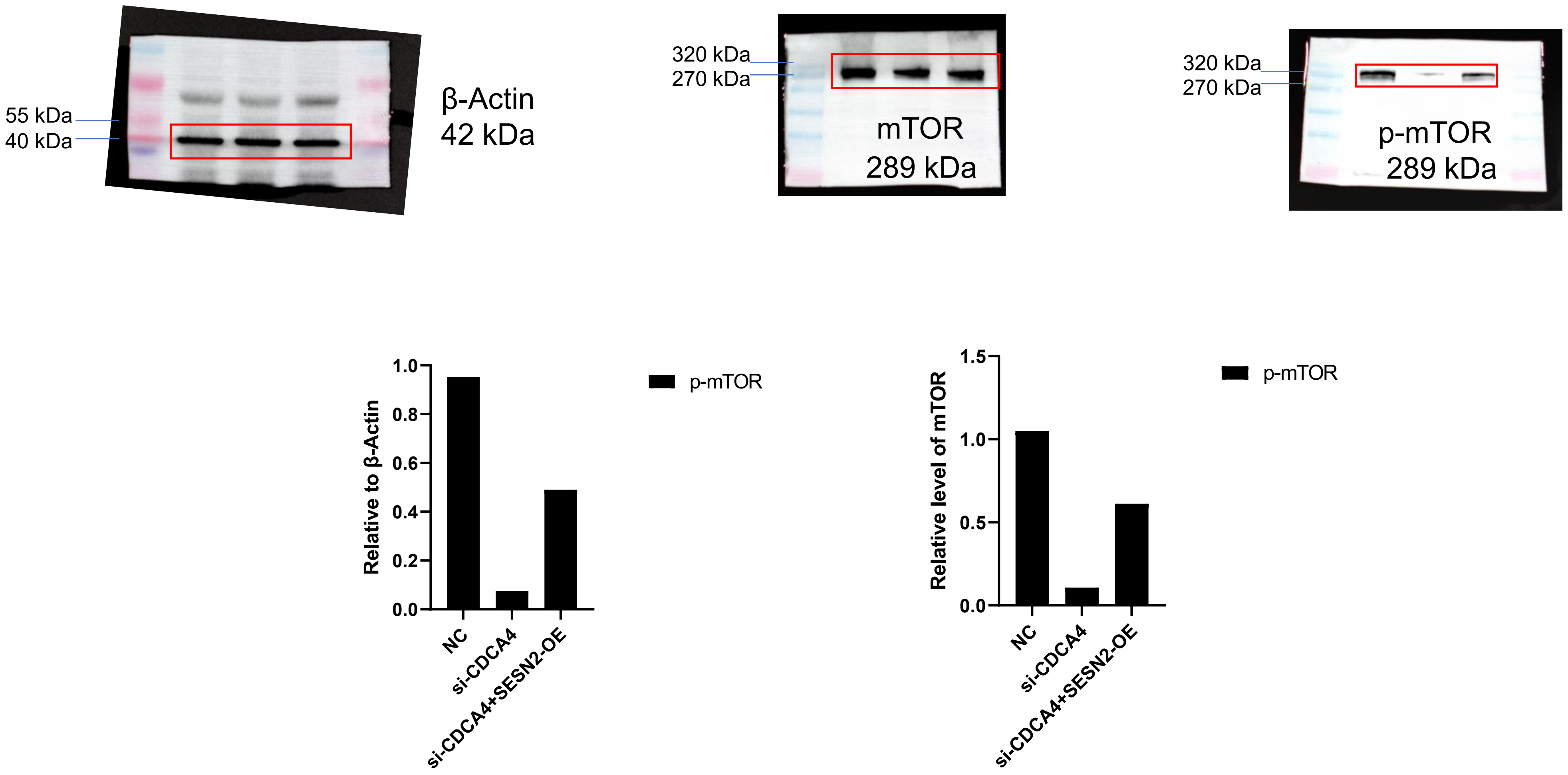

Supplement: Supplementary file 1 [file cancers-18-02517-s001.zip › cancers-4394988-Supplementary File S2.pdf]
