# Supplementary material for: CDCA4 Promotes Lipid Metabolism in Triple-Negative Breast Cancer Through Activation of the SESN2/mTOR/SREBP1 Pathway
Source: Cancers (Basel). 2026 Aug 6;18(15):2517. doi: 10.3390/cancers18152517 (PMC13465203; doi:10.3390/cancers18152517)
Supplement: Supplementary file 1 [file cancers-18-02517-s001.zip › cancers-4394988-Supplementary.pdf]

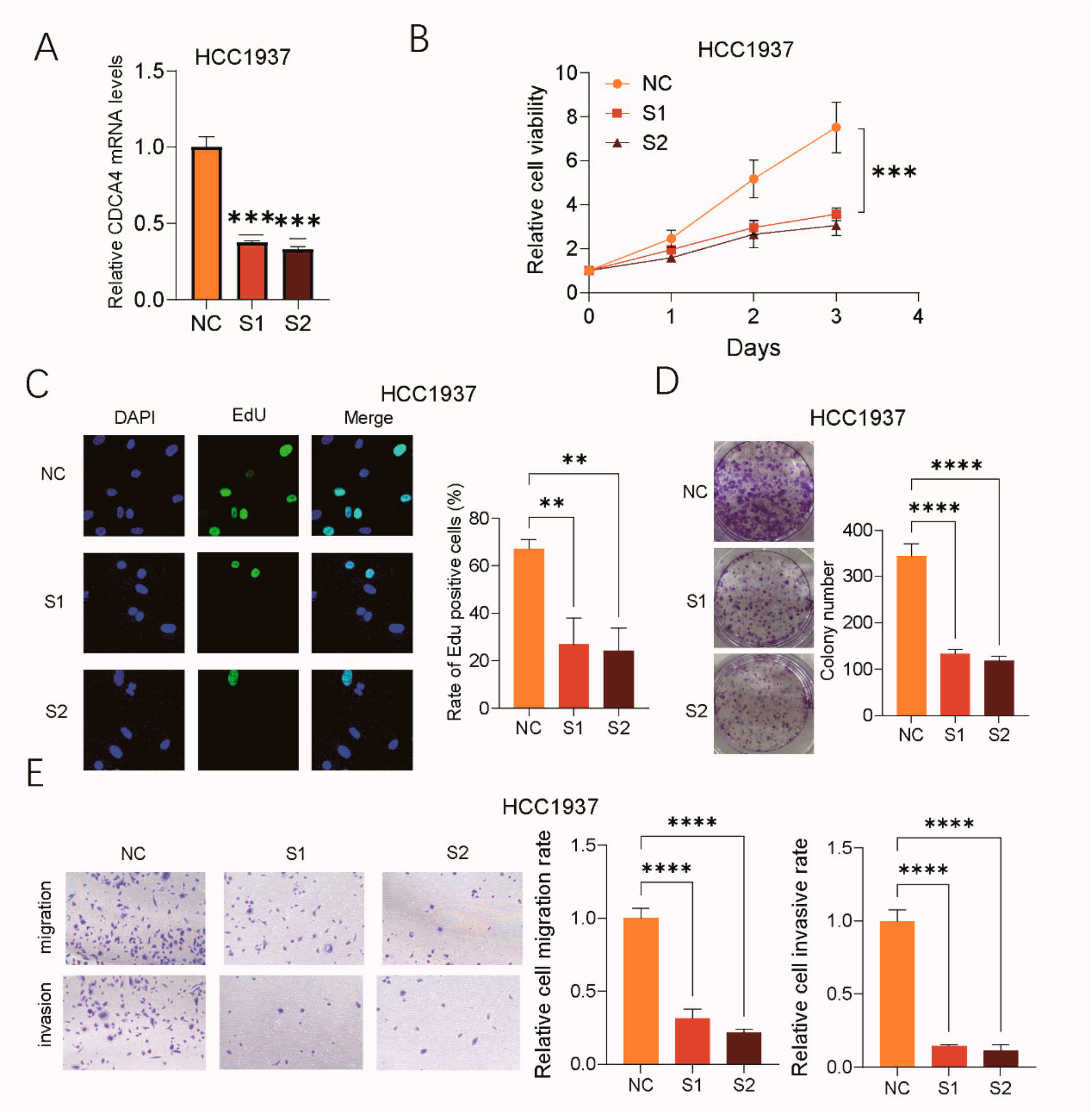

Figure S1. Functional analyses of CDCA4 knockdown in HCC1937 cells. (A) Western blot and RT-qPCR analysis of CDCA4 expression in HCC1937 cells after transfection with siRNAs targeting CDCA4. (B) MTS assay in HCC1937 cells following CDCA4 knockdown. (C) EdU incorporation assay in HCC1937 cells following CDCA4 knockdown. (D) Colony formation assay in HCC1937 cells following CDCA4 knockdown. (E) Transwell migration and invasion assays in HCC1937 cells following CDCA4 knockdown.

 https://doi.org/10.3390/cancers18152517

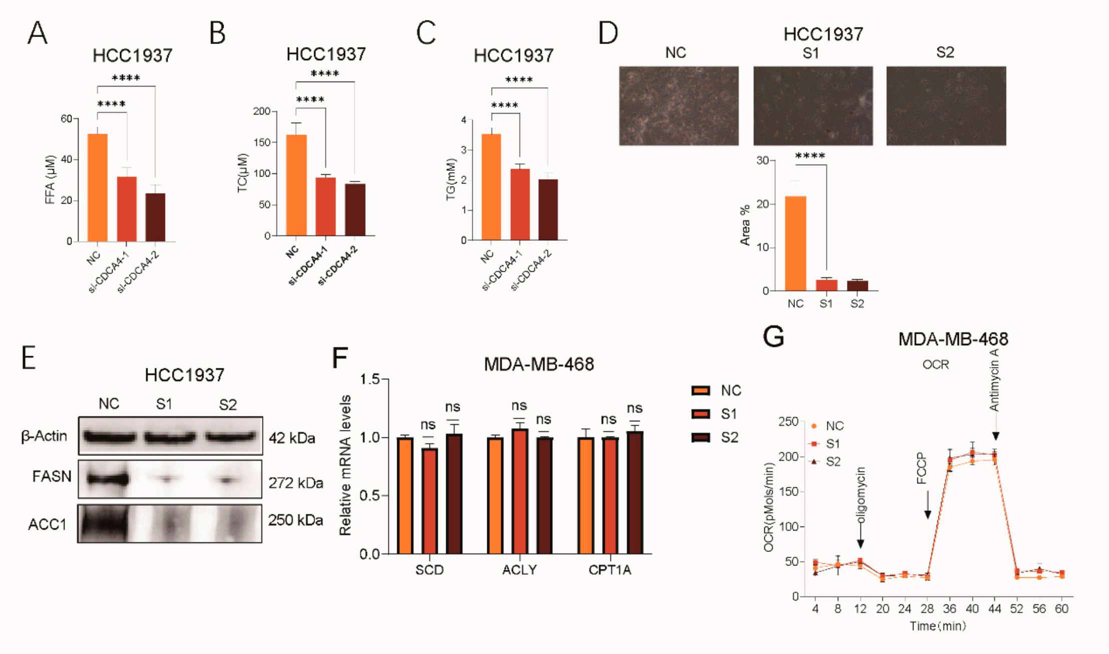

Figure S2. Lipid metabolic analyses in HCC1937 cells after CDCA4 knockdown. (A-D) Quantification of intracellular free fatty acid (FFA) levels (A), total cholesterol (TC) content (B), triglyceride (TG) content (C), and Oil Red O staining (D) in control and CDCA4-knockdown HCC1937 cells. (E) Western blot analysis of FASN and ACC1 protein expression in control and CDCA4-knockdown HCC1937 cells. (F) RT-qPCR analysis of SCD1, ACLY, and CPT1A mRNA levels in control and CDCA4-knockdown MDA-MB-468 cells. (G) Oxygen consumption rate (OCR) analysis in control and CDCA4-knockdown MDA-MB-468 cells.

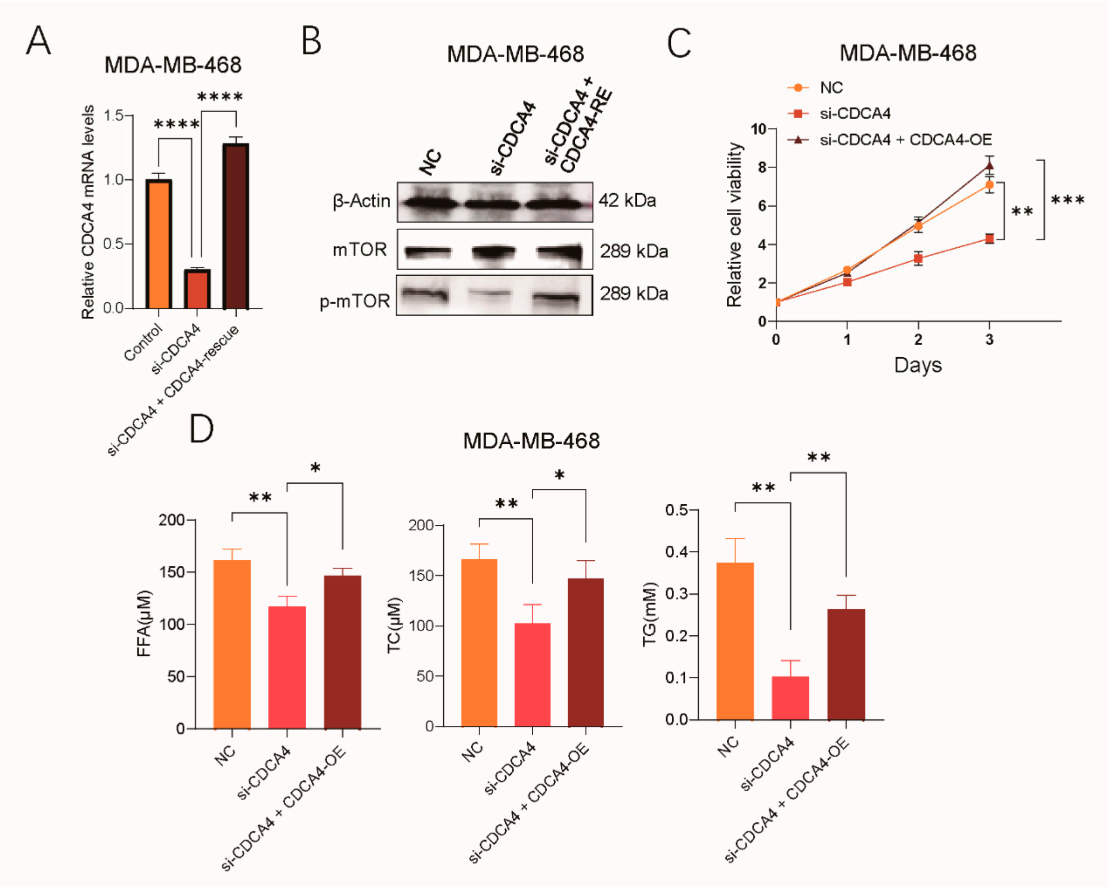

Figure S3. Rescue of CDCA4 expression reverses the effects of CDCA4 knockdown. (A) RT-qPCR analysis of CDCA4 mRNA expression in control cells, CDCA4-knockdown cells, and CDCA4-knockdown cells with CDCA4 re-expression. (B) Western blot analysis of mTOR and p-mTOR protein levels in control cells, CDCA4-knockdown cells, and CDCA4-knockdown cells with CDCA4 re-expression. (C) Cell viability analysis of control cells, CDCA4-knockdown cells, and CDCA4-knockdown cells with CDCA4 re-expression. (D) Quantification of intracellular free fatty acid (FFA), total cholesterol (TC), and triglyceride (TG) levels in control cells, CDCA4-knockdown cells, and CDCA4-knockdown cells with CDCA4 re-expression.

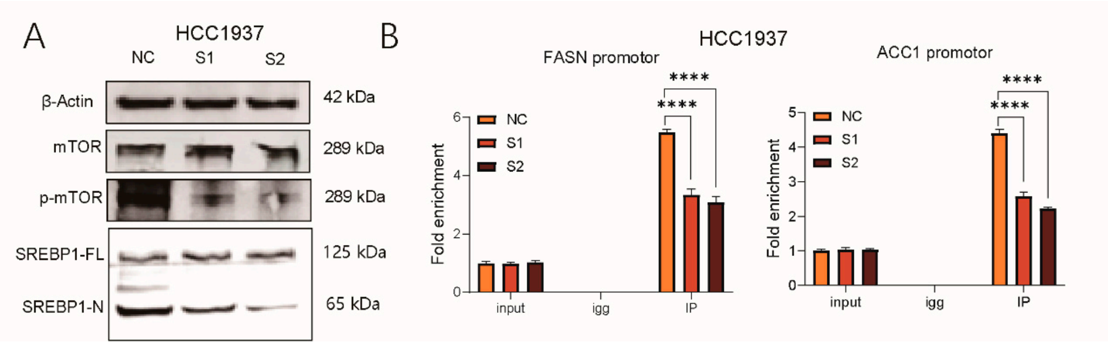

Figure S4. CDCA4 knockdown affects mTOR/SREBP1 signaling in HCC1937 cells. (A) Western blot analysis of mTOR pathway activation and SREBP1-N expression in HCC1937 cells following CDCA4 knockdown. (B) ChIP-qPCR analysis of SREBP1-N occupancy at the FASN and ACC1 promoter regions in HCC1937 cells following CDCA4 knockdown.

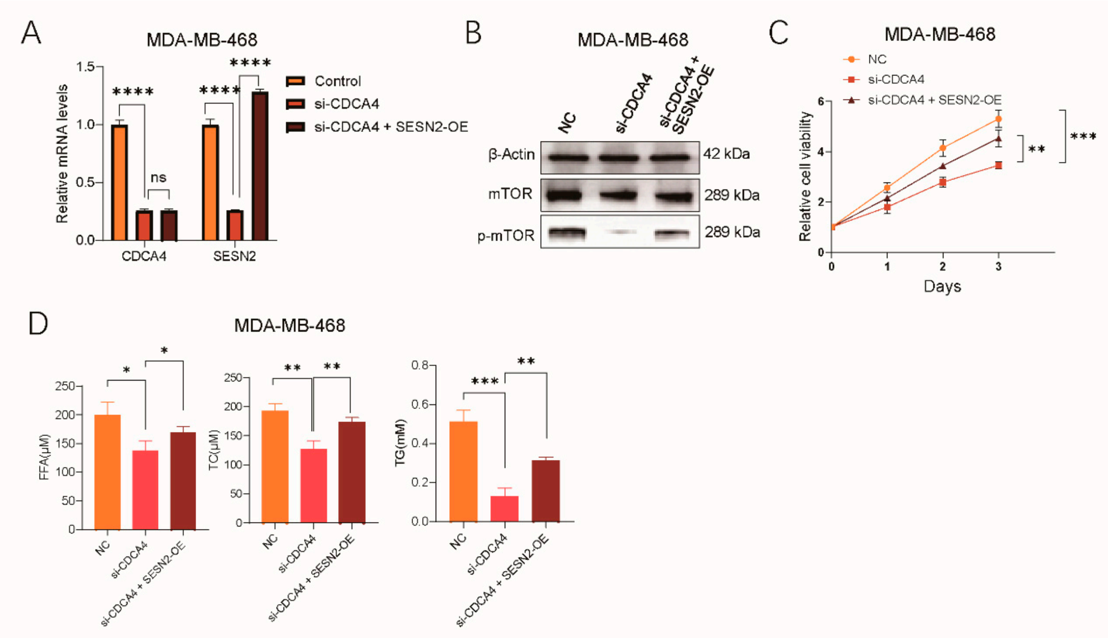

Figure S5. SESN2 overexpression partially rescues the effects of CDCA4 knockdown in MDA-MB-468 cells. (A) RT-qPCR analysis of CDCA4 and SESN2 mRNA levels in control cells, CDCA4-knockdown cells, and CDCA4-knockdown cells with SESN2 overexpression. (B) Western blot analysis of mTOR and p-mTOR protein levels in control cells, CDCA4-knockdown cells, and CDCA4-knockdown cells with SESN2 overexpression. β-Actin was used as the loading control. (C) Cell viability analysis in control cells, CDCA4-knockdown cells, and CDCA4-knockdown cells with SESN2 overexpression. (D) Quantification of intracellular free fatty acid (FFA), total cholesterol (TC), and triglyceride (TG) levels in control cells, CDCA4-knockdown cells, and CDCA4-knockdown cells with SESN2 overexpression.

Supplementary Table S1 Sequences of oligonucleotides used in this study		
oligonucleotide name	oligonucleotide sequences (5'-3')	oligonucleotide sequences (3'-5')
RT-PCR- CDCA4	CGCGGGTCAGGACACAAT	CAGTGCTCACGTGTCAATGC
RT-PCR- SESN2	TCTTACCTGGTAGGCTCCCAC	AGCAACTTGTTGATCTCGCTG
RT-PCR- β -actin	AGAGCGAGGAGGACAAGTTC	CGCTCTCGTTCAGAAGTCTC
oligonucleotide name	Target sequence	
siCDCA4-1	UAGGUUGAAGUCUAGAAUGAATT	
siCDCA4-2	UUAUCUGGUGGUGCUUUCUAATT	
siSESN2-1	AGGAGGGAGUAUUAGAUUAUATT	
si SESN2-2	AGGGCAUGUGAUGACUGUAAATT	

Antibody		Cat No.
mTOR Monoclonal antibody	Proteintech	66888-1-Ig
Phospho-mTOR (Ser2448) Monoclonal antibody	Proteintech	67778-1-Ig
Beta Actin Recombinant antibody	Proteintech	81115-1-RR
ACC1 Polyclonal antibody	Proteintech	21923-1-AP
FASN Polyclonal antibody	Proteintech	10624-2-AP-50ul
CDCA4 Polyclonal antibody	Proteintech	11625-1-AP
Sestrin 2 Polyclonal antibody	Proteintech	10795-1-AP

https://doi.org/10.3390/cancers18152517
